# Transcriptional response to prolonged perchlorate exposure in the methanogen *Methanosarcina barkeri* and implications for Martian habitability

**DOI:** 10.1038/s41598-021-91882-0

**Published:** 2021-06-11

**Authors:** Rachel L. Harris, Andrew C. Schuerger, Wei Wang, Yuri Tamama, Zachary K. Garvin, Tullis C. Onstott

**Affiliations:** 1 Department of Geosciences, Princeton University, Princeton, NJ USA; 2 Department of Plant Pathology, University of Florida, Gainesville, FL USA; 3 Lewis-Sigler Institute for Integrative Genomics, Department of Molecular Biology, Princeton University, Princeton, NJ USA; 4 Department of Organismic and Evolutionary Biology, Harvard University, Cambridge, MA USA

**Keywords:** Astrobiology, Gene regulatory networks, Genome informatics, Applied microbiology, Archaea, Biogeochemistry, Environmental microbiology, Microbial ecology, Carbon cycle, Inner planets, Biogeochemistry

## Abstract

Observations of trace methane (CH_4_) in the Martian atmosphere are significant to the astrobiology community given the overwhelming contribution of biological methanogenesis to atmospheric CH_4_ on Earth. Previous studies have shown that methanogenic *Archaea* can generate CH_4_ when incubated with perchlorates, highly oxidizing chaotropic salts which have been found across the Martian surface. However, the regulatory mechanisms behind this remain completely unexplored. In this study we performed comparative transcriptomics on the methanogen *Methanosarcina barkeri*, which was incubated at 30˚C and 0˚C with 10–20 mM calcium-, magnesium-, or sodium perchlorate. Consistent with prior studies, we observed decreased CH_4_ production and apparent perchlorate reduction, with the latter process proceeding by heretofore essentially unknown mechanisms. Transcriptomic responses of *M. barkeri* to perchlorates include up-regulation of osmoprotectant transporters and selection against redox-sensitive amino acids. Increased expression of methylamine methanogenesis genes suggest competition for H_2_ with perchlorate reduction, which we propose is catalyzed by up-regulated molybdenum-containing enzymes and maintained by siphoning diffused H_2_ from energy-conserving hydrogenases. Methanogenesis regulatory patterns suggest Mars’ freezing temperatures alone pose greater constraints to CH_4_ production than perchlorates. These findings increase our understanding of methanogen survival in extreme environments and confers continued consideration of a potential biological contribution to Martian CH_4_.

## Introduction

**Figure 1:**
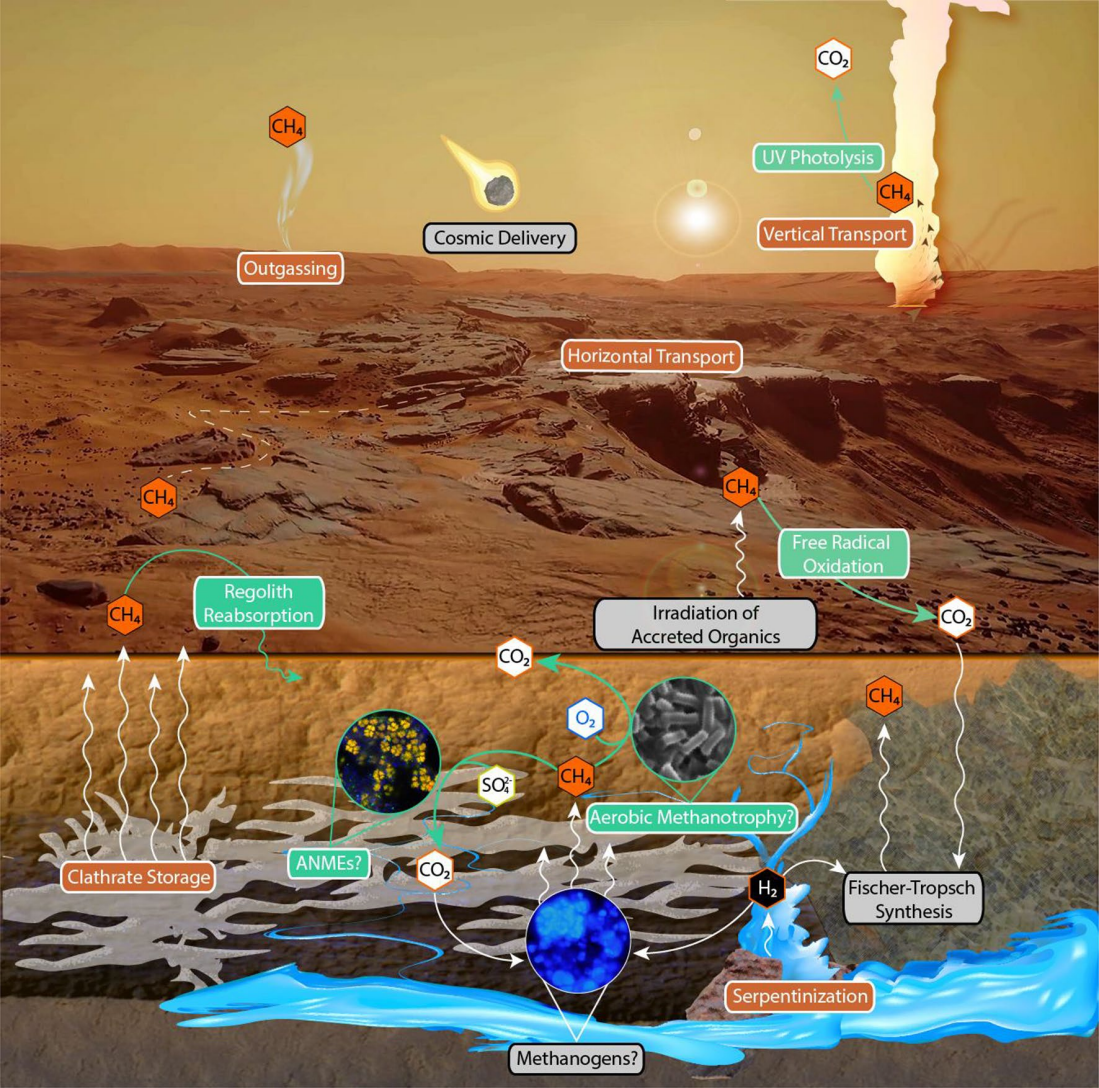
Proposed model for Martian CH_4_ cycle. Sources indicated by grey boxes. Sinks are highlighted by green boxes and accompanying arrows. Reservoirs, transport systems, and substrate-generating intermediates are denoted by orange boxes. Speculative biological reservoirs are indicated via question mark [?]. This figure was generated using images from *Mars Reconnaissance Orbiter* and the *Mars 2030* virtual reality game (produced by NASA in collaboration with the MIT AeroAstro Lab and Fusion Media Group, Doral, FL) in accordance with the fair use doctrine of United States copyright law. Abbreviations: ANMEs, anaerobic methanotrophs.

The story of Martian atmospheric methane (CH_4_) remains enigmatic and under intense debate. In the past 15 years, a growing body of evidence has unfolded to suggest episodic appearances (and disappearances) of ppbv-level CH_4_^1–7^. Myriad abiotic mechanisms have been suggested as potential CH_4_ sources, including cometary impacts^8^, UV degradation of meteoritic and interplanetary dust particle organics^9–11^, and Fischer–Tropsch-type synthesis in the subsurface coupled to either serpentinization of ultramafic silicates^12,13^ or radiolysis of H_2_O^14^, which subsequently releases CH_4_ to the surface through seeps and salinity-induced hydrate dissociation^15^ (Fig. 1). On Earth, however, nearly 70% of CH_4_ is of biological origin, generated by methanogenic *Archaea*^16^. This has led to an extensive debate considering the biological origin of Martian CH_4_. Understandably, the quest to comprehend the nature of CH_4_ cycling on Mars is a fervent one, as it may be the most conspicuous biosignature detected on Mars to date. In the absence of returned Martian samples or stable isotopic data of Martian atmospheric CH_4_, presently the best approach to constrain this debate is to experimentally test the ability of methanogenic *Archaea* to metabolize under Martian conditions.

Methanogens are not only among the most deeply rooted microorganisms in the tree of life, but they have also proliferated into nearly every habitable anaerobic environment and possess conserved adaptions for growth and survival under exposure to prolonged desiccation^17–19^, high salinity^20–24^, strong oxidants^25,26^, and extremes in temperature, pH, and pressure^27–31^. Thus, they have been the subject of extensive study to infer how hostile conditions simulating modern Mars may allow—or inhibit—biological methanogenesis^17,19,25,26,31–34^.

Perchlorate salts are highly soluble, chaotropic compounds of primarily human-manufactured origin on Earth^35^, with naturally occurring deposits mostly limited to evaporites in hyper-arid regions such as the Atacama Desert^36^ and the Antarctic Dry Valleys^37^. On Mars, however, perchlorates appear to be pervasive^38–44^. Perchlorates are of great interest to astrobiology—particularly, Martian habitability studies—for their hygroscopicity and low eutectic temperatures, which allows for the formation of stable liquid water brines at temperatures as low as -74.6˚C and 55% relative humidity^45–50^. Previous work has reported decreased CH_4_ production in methanogenic cultures supplemented with increasing concentrations of perchlorate salts^25,26^. This suggests that this aspect of the Martian environment may be debilitating to any potential biological methanogenesis that could occur under favorable conditions, but the mechanisms resulting in this apparent inhibition in analogue experiments have not been identified. Here we utilize transcriptomics to evaluate regulatory responses of the methanogenic archaeon *Methanosarcina barkeri* strain MS during a 28-day exposure to high concentrations of sodium-, magnesium-, and calcium perchlorate salts at 30 °C and 0 °C to assess if perchlorate salts are inhibitory to methanogenesis—and if so, how—in order to appraise the potential for methanogens’ survival on Mars.

## Results

### Transcriptome assembly statistics

*M. barkeri* possesses the second largest described genome amongst the methanogenic *Archaea*^51^. This genome comprises a 4.53 megabase (Mb) circular chromosome and a 40 kilobase (kb) plasmid, which collectively encode 3,760 genes, 3,470 of which are protein-encoding coding sequence (CDS) regions (3.17 Mb). RNA sequencing yielded a total of 1,500,716,043 quality paired end reads across 24 libraries (8 conditions × 3 replicates/condition) with a mean Phred (sequence quality) score of 36. On average 1.6 ± 0.7% of 30 °C and 1.5 ± 0.5% of 0 °C quality-filtered reads mapped back to CDS regions (n = 12 libraries/temperature condition; Table S1 consistent with expectations that mRNA typically comprises 1–5% of total RNA in prokaryotic cells^52^. Average fragment counts per million mapped reads (FPM) are organized by gene position in Table S2 and visualized in Figs. S1–S2.

### Methanogenesis and associated regulatory responses

**Figure 2:**
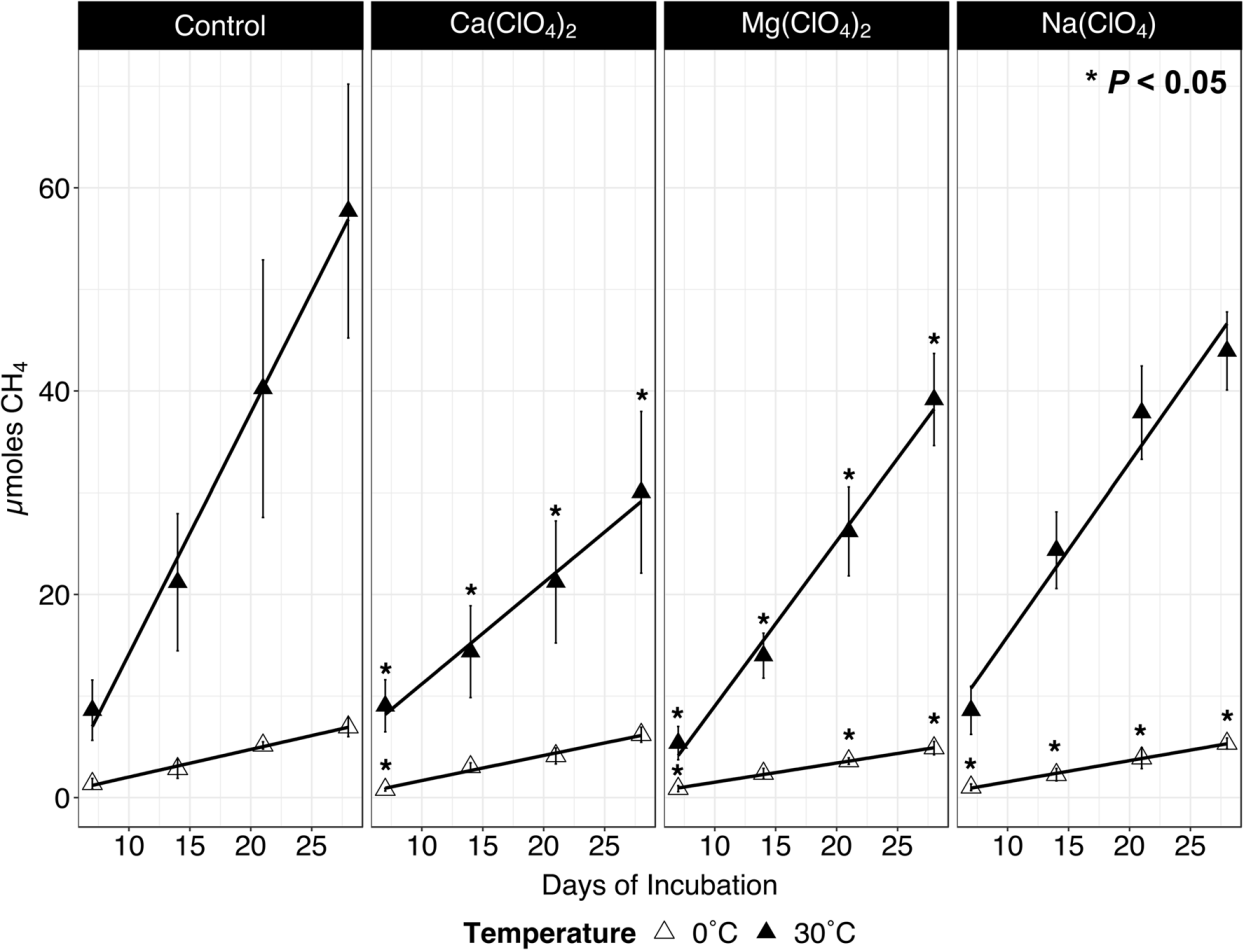
Cumulative CH_4_ production by *M. barkeri* strain MS (from left to right) in media without perchlorate (Control), Ca(ClO_4_), Mg(ClO_4_)_2_, or Na(ClO_4_) at 30 °C (filled triangles) and 0 °C (open triangles). Statistically significant differences in CH_4_ production in perchlorate-supplemented incubations relative to the control of the same temperature are indicated by asterisks (*) (paired t-test, *P* < 0.05).

At 30 °C, the addition of Ca(ClO_4_)_2_, Mg(ClO_4_)_2_, and Na(ClO_4_) reduced total net CH_4_ production by 48%, 32%, and 24%, respectively, relative to the perchlorate-free control (Fig. 2). Significant reduction in CH_4_ production rates were observed across all conditions at 0 °C with respect to their 30 °C counterparts, and each perchlorate condition at 0 °C showed a statistically significant decrease in CH_4_ for at least one time point relative to the 0 °C control (Fig. 2). No CH_4_ production was observed in the media blank controls (data not shown). Cultures were monitored via optical density measurements at 600 nm (OD_600_), but perchlorate-amended media experienced precipitate formation which made obtaining reliable growth data difficult (Fig. S3).

**Figure 3:**
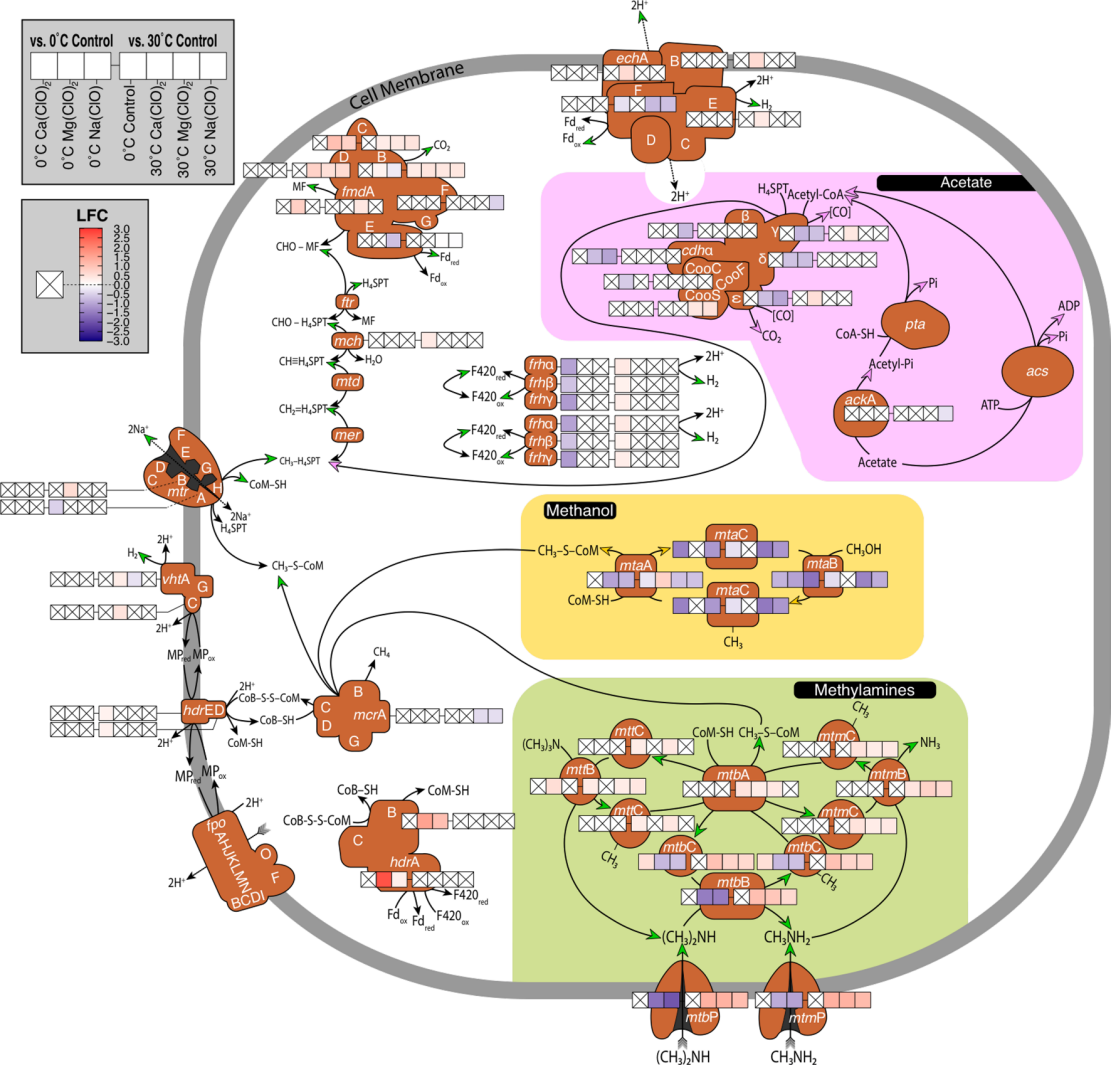
Statistically significant differential expression (Log_2_-fold change, LFC; Wald test, *P* < 0.05) of methanogenesis genes in *M. barkeri* strain MS. Differential expression of each gene across 7 assessed conditions is indicated via heatmap. Averages are presented for multi-copy genes (for exact values, reference Tables 1 and 2). Differential expression of genes in 0 °C perchlorate-amended transcriptomes are relative to the expression patterns of the 0 °C perchlorate-free control. Perchlorate-amended 30 °C and 0 °C perchlorate-free control transcriptomes are relative to the expression patterns of the 30 °C perchlorate-free control. Crossed white boxes indicate no statistically significant difference in expression. Heatmaps are excluded from genes exhibiting no significant differential expression across all conditions. Genes involved in non-hydrogenotrophic (H_2_/CO_2_) pathways are encompassed in colored boxes with pathway directionality indicated by matching arrowheads (green: methylamines; pink: acetate; yellow: methanol). Full names of listed genes and metabolites are found in Tables S13 and S14, respectively.

When incubated at 0 °C, the perchlorate-free control demonstrated significant up-regulation of several genes in the hydrogenotrophic pathway relative to the 30 °C perchlorate-free control (Table 1, Fig. 3), including log_2_-fold changes (LFC) in molybdenum (Mo)-formylmethanofuran dehydrogenase subunit B (*fmd*B), methenyl-tetrahydrosarcinapterin (H_4_SPT) cyclohydrolase (*mch*), and periplasmic heterodisulphide reductase (*hdr*DE). Likewise, significant down-regulation was observed in the sodium ion (Na^+^) transporter methyl-H_4_SPT:coenzyme M methyltransferase complex (*mtr*A), as well as the F_420_-reducing subunit of the periplasmic energy conserving hydrogenase (*ech*F). All subunits of both coenzyme F_420_ hydrogenases (*frh*αβγ) were significantly up-regulated in perchlorate-free 0 °C incubations (Table 1, Fig. 3). In the perchlorate-treated incubations at 0 °C, we only observed down-regulation of *frh*αβγ in the presence of Ca(ClO_4_)_2_ with respect to the 0 °C perchlorate-free control (Table 2, Fig. 3).

**Table 1:** Differential expression (Log_2_-fold change ± standard error) of *M. barkeri* MS genes implicated in the Wood-Ljungdahl pathway and hydrogenotrophic methanogenesis.

Gene	0 °C ClO_4_^–^free	30 °C Ca(ClO_4_)_2_	30 °C Mg(ClO_4_)_2_	30 °CNa(ClO_4_)
*fmd*A	n.s.d	n.s.d	0.40 ± 0.10 (W)	n.s.d
			0.40 ± 0.10 (W)	
*fmd*B	0.34 ± 0.12 (W)	0.44 ± 0.14 (Mo)	0.44 ± 0.14 (Mo)	0.44 ± 0.14 (Mo)
			0.36 ± 0.13 (Mo)	0.38 ± 0.13 (Mo)
*fmd*C	n.s.d	0.32 ± 0.14 (Mo)	0.39 ± 0.13 (Mo)	0.46 ± 0.13 (Mo)
		0.32 ± 0.14 (Mo)	0.39 ± 0.13 (Mo)	0.45 ± 0.13 (Mo)
*fmd*D	n.s.d	0.75 ± 0.15 (Mo)	0.52 ± 0.15 (Mo)	0.59 ± 0.15 (Mo)
		0.75 ± 0.15 (Mo)	0.52 ± 0.15 (Mo)	0.59 ± 0.15 (Mo)
*fmd*E	n.s.d	n.s.d	0.51 ± 0.12 (Mo)	0.43 ± 0.13 (Mo)
			− 0.45 ± 0.19 (Mo)	− 0.50 ± 0.19 (Mo)
*ech*A	n.s.d	0.62 ± 0.14	n.s.d	n.s.d
*ech*B	n.s.d	0.60 ± 0.19	n.s.d	n.s.d
*ech*E	n.s.d	0.35 ± 0.12	n.s.d	n.s.d
*ech*F	− 0.33 ± 0.15	n.s.d	− 0.77 ± 0.20	− 0.70 ± 0.20
*mch*	0.21 ± 0.08	n.s.d	n.s.d	n.s.d
*hdr*D	0.23 ± 0.07	n.s.d	n.s.d	n.s.d
*hdr*E	0.22 ± 0.09	n.s.d	n.s.d	n.s.d
*mtr*A	− 0.51 ± 0.22	n.s.d	n.s.d	n.s.d
*mtr*B	n.s.d	0.59 ± 0.15	n.s.d	n.s.d
*vht*A	n.s.d	0.31 ± 0.12	− 0.32 ± 0.12	n.s.d
*vht*C	n.s.d	0.46 ± 0.17	n.s.d	n.s.d
*frh*α	0.38 ± 0.12	n.s.d	n.s.d	n.s.d
	0.35 ± 0.12			
*frh*β	0.31 ± 0.13	n.s.d	n.s.d	n.s.d
	0.17 ± 0.07			
*frh*γ	0.30 ± 0.13	n.s.d	n.s.d	n.s.d
	0.29 ± 0.13			
*mcr*A	n.s.d	n.s.d	− 0.40 ± 0.16	− 0.36 ± 0.16
*coo*S	n.s.d	n.s.d	0.40 ± 0.13	0.49 ± 0.13
*cdh*ε	n.s.d	0.53 ± 0.14	n.s.d	n.s.d
*cdh*γ	n.s.d	0.39 ± 0.16	n.s.d	n.s.d

LFC is relative to 30 °C perchlorate-free control. Multiple entries indicate statistical significance in multi-copy genes (Wald test, *p* < 0.05). Abbreviations: n.s.d., no significant difference from control; W, tungsten active site; Mo, molybdenum active site. If gene is not listed, no significant differential expression was observed across all treatments. Full names of genes are referenced in the text and are also listed in Table S12.

**Table 2:** Differential expression (Log_2_-fold change ± standard error) of *M. barkeri* MS genes implicated in the Wood-Ljungdahl pathway and hydrogenotrophic methanogenesis.

Gene	0 °C Ca(ClO_4_)_2_	0 °C Mg(ClO_4_)_2_	0 °C Na(ClO_4_)
*fmd*A	n.s.d	0.79 ± 0.23 (W)	n.s.d
*fmd*B	n.s.d	1.04 ± 0.24 (W)	0.72 ± 0.24 (W)
		− 0.65 ± 0.22 (Mo)	− 0.66 ± 0.22 (Mo)
			− 0.74 ± 0.23 (Mo)
*fmd*C	n.s.d	1.05 ± 0.32 (W)	0.76 ± 0.33 (W)
*fmd*E	n.s.d	n.s.d	− 0.69 ± 0.22 (Mo)
*hdr*A	n.s.d	2.66 ± 0.71	1.23 ± 0.89
			− 0.69 ± 0.29
*hdr*B	n.s.d	1.52 ± 0.54	1.06 ± 0.61
*cdh*ε	n.s.d	− 0.71 ± 0.22	− 1.00 ± 0.21
*cdh*γ	n.s.d	− 0.82 ± 0.19	− 0.67 ± 0.19
		− 0.82 ± 0.22	− 0.71 ± 0.22
*cdh*α	n.s.d	− 0.68 ± 0.22	− 1.01 ± 0.22
		− 0.77 ± 0.23	− 1.04 ± 0.22
*cdh*β	n.s.d	n.s.d	− 0.76 ± 0.22
			− 0.76 ± 0.22
*cdhδ*	n.s.d	− 0.66 ± 0.21	− 0.71 ± 0.20
		− 0.67 ± 0.20	− 0.72 ± 0.20

LFC is for 0 °C and perchlorate-incubated *M. barkeri* MS relative to 0 °C perchlorate-free control. (Wald test, *p* < 0.05). Abbreviations: n.s.d., no significant difference from control; W, tungsten active site; Mo, molybdenum active site. If gene is not listed, no significant differential expression was observed across all treatments. Full names of genes are referenced in the text and are also listed in Table S12.

At 30 °C, methanogenesis-associated regulatory responses were shared across all perchlorate conditions (Table 1). This included up-regulation of B, C, and D subunits of (Mo)-formylmethanofuran dehydrogenase, *fmd*, which catalyzes the CO_2_ reduction step of the hydrogenotrophic pathway (Fig. 3). In the presence of both Mg(ClO_4_)_2_ and Na(ClO_4_), one copy of (Mo)-*fmd*E exhibited significant up-regulation, while the other was significantly down-regulated (Table 1)_._ Unique to the Mg(ClO_4_)_2_ incubations was the up-regulation of tungsten (W)-*fmd*A. In contrast, perchlorate-supplemented treatments incubated at 0 °C demonstrated statistically significant down-regulation of Mo-*fmd* genes, but up-regulation of the (W)-*fmd* operon (Table 2).

Energy conserving hydrogenase subunit F, *ech*F, which supplies reduced ferredoxin to *fmd*, was down-regulated in the presence of Mg(ClO_4_)_2_ and Na(ClO_4_) at 30 °C, whereas most subunits of *ech* were significantly up-regulated in 30 °C Ca(ClO_4_)_2_ conditions (Fig. 3). Among other hydrogenases demonstrating significant up-regulation was methanophenazine-dependent hydrogenase, *vht*, specifically, the large subunit *vht*A and cytochrome b subunit *vht*C (Fig. 3). At 0 °C, *ech* hydrogenases were not differentially regulated in the presence of perchlorates relative to the perchlorate-free control (Fig. 3).

Despite H_2_ being the only reducing equivalent provided for the production of CH_4_ in our incubations, the addition of Ca(ClO_4_)_2_, Mg(ClO_4_)_2_, and Na(ClO_4_) resulted in significant up-regulation of all genes in the mono-, di-, and trimethylamine pathways and associated membrane permeases in the 30 °C perchlorate treatments (Fig. 3, Tables S7, S9, S11).

The terminal step encoding methyl-coenzyme M (CH_3_-CoM) reductase subunit alpha (*mcr*A) was down-regulated at 30 °C in Mg(ClO_4_)_2_ and Na(ClO_4_) enrichments (Fig. 3, Table 1). Although this reduction in expression is consistent with the decreased CH_4_ production rates observed in these treatments (Fig. 2), the Ca(ClO_4_)_2_-amended *M. barkeri* MS, which generated the least CH_4_, showed no significant difference in expression of the *mcr* complex relative to the 30 °C perchlorate-free control (Fig. 3, Table 1). Furthermore, no elements of *mcr* were significantly differentially expressed at 0 °C in the perchlorate-free control relative to the 30 °C perchlorate-free control (Fig. 3) despite its far lower CH_4_ production rate (Fig. 2). Therefore, the decreased expression of *mcr*A does not appear to be sufficient to explain the associated decrease in CH_4_ production rates with perchlorate exposure at 30 °C or incubation at 0 °C.

The carbon monoxide dehydrogenase/acetyl-CoA synthase complex (CODH/ACS), which plays a key role in both energy conversation and carbon fixation via the Wood-Ljungdahl pathway, demonstrated significant regulatory changes as a function of perchlorate exposure and temperature. The *coo*S subunit of CODH, which reversibly converts CO and CO_2_, was up-regulated in 30 °C Mg(ClO_4_)_2_ and Na(ClO_4_) treatments (Fig. 3, Table 1). Carbon monoxide dehydrogenase subunit epsilon (*cdh*ε), which recycles ferredoxin in the reversible conversion between CO and CO_2_, was up-regulated with Ca(ClO_4_)_2_ at 30 °C (Fig. 3, Table 1), but was down-regulated in the 0 °C Mg(ClO_4_)_2_ and Na(ClO_4_) treatments (Fig. 3, Table 2). The 30 °C Ca(ClO_4_)_2_ treatment also demonstrated significant up-regulation of 5-H_4_SPT:corrinoid Fe-S protein methyltransferase (*cdh*γ), which plays a key role in the generation H_4_SPT and acetyl-CoA for biomass synthesis in the Wood-Ljungdahl pathway (Fig. 3, Table 1). Both copies of this gene were down-regulated at 0 °C in the Mg(ClO_4_)_2_ and Na(ClO_4_) treatments (Fig. 3, Table 2).

Both alpha and beta chains of ACS, respectively encoded by *cdh*α and *cdh*β, were down-regulated in the 0 °C Na(ClO_4_) treatment with respect to the 0 °C perchlorate-free control, while only *cdh*α was down-regulated in the 0 °C Mg(ClO_4_)_2_ treatment (Fig. 3, Table 2). *Cdh*δ, which encodes an iron-sulfur corrinoid protein, was also down-regulated at 0 °C in Mg(ClO_4_)_2_ and Na(ClO_4_)-supplemented incubations (Fig. 3, Table 2). We observed no significant differential expression of CODH/ACS complex genes in the 0 °C Ca(ClO_4_)_2_ treatment (Fig. 3).

### Concurrent up-regulation of ammonium transporters, Mo-nitrogenases, and P-II repressors

**Figure 4:**
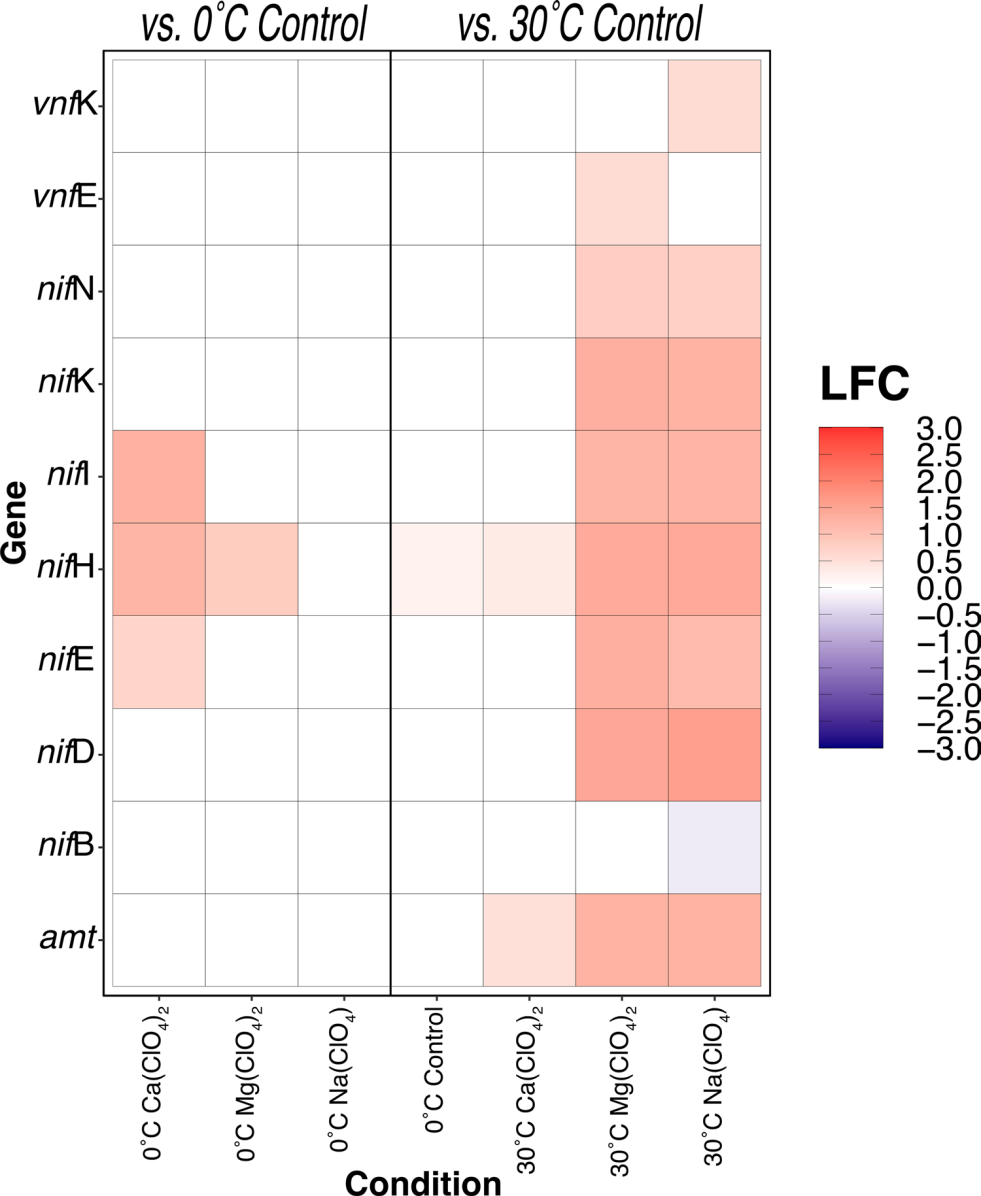
Differential expression (Log_2_-fold change, LFC) of ammonium transporters (*amt*), Mo-containing nitrogenase (*nif*), and V-containing nitrogenase genes in *M. barkeri* MS. Perchlorate-amended 30 °C and 0 °C perchlorate-free control cultures are relative to 30 °C perchlorate-free control. 0 °C perchlorate-amended cultures are relative to 0 °C perchlorate-free control. Significance identified via Wald test (*P* < 0.05). Full names of proteins encoded by listed genes are found in Table S15.

The most substantial up-regulation patterns were observed in genes relating to nitrogen cycling in perchlorate-incubated treatments. The 30 °C Mg(ClO_4_)_2_ and Na(ClO_4_)-amended conditions showed positive differential expression of 6 genes in the (Mo)-nitrogenase (hereafter (Mo)-Nase) complex (Fig. 4) including *nif*H, the MoFe-dinitrogen reductase that is responsible for electron transfer to the α_2_β_2_ N_2_ binding site (encoded by *nif*D and *nif*K, respectively) via ATP hydrolysis, as well as the P-II regulatory repressors, *nif*I, which shut off N_2_ fixation when ammonia is bioavailable^53–56^. Biosynthesis and assembly proteins for (Mo)-Nase, *nif*E and *nif*N, were also upregulated at 30 °C with Mg(ClO_4_)_2_ and Na(ClO_4_). At 30 °C, only *nif*H demonstrated significant up-regulation in both the Ca(ClO_4_)_2_ and perchlorate-free control, but the 0 °C Ca(ClO_4_)_2_ treatment also showed significant up-regulation of *nif*I and *nif*E (Fig. 4).

We observed substantial up-regulation of ammonium transporters (*amt*) in the 30 °C perchlorate treatments, but no significant differences in *amt* expression were observed in any 0 °C treatments (Fig. 4). Ammonium measurements confirmed that bioavailable fixed N remained replete (mM-level concentration) across all conditions for the duration of the incubation (Table S3).

### Up-regulation of osmostress response genes

In the 30 °C Mg(ClO_4_)_2_ treatment, the complete operon for osmostress protectants uptake A, *opuA*, observed positive LFC of 0.64 ± 0.17 (*opuA*A, *P* < 0.001), 0.73 ± 0.20 (*opuA*B, *P* < 0.001), and 0.66 ± 0.30 (*opuA*C, *P* = 0.02). In the 30 °C Na(ClO_4_) treatment, significant up-regulation was also observed for *opuA*A (LFC = 0.54 ± 0.17, *P* = 4 $\times$ 10^–3^) and *opuA*B (LFC = 0.59 ± 0.20, *P* = 7 $\times$ 10^–3^). *OpuA* is responsible for the uptake of extracellular glycine betaine, belonging to a family of ABC transporters that hydrolyze ATP to import glycine betaine and other osmoprotectants such as proline^57–59^. The *opu* family also includes uptake systems for choline, a glycine betaine precursor^59^, but *M. barkeri* lacks the cellular machinery for de novo glycine betaine synthesis^60^. Relative to the 0 °C perchlorate-free control, significant down-regulation of *opuA*A in 0 °C Mg(ClO_4_)_2_ (LFC = − 0.94 ± 0.30, *P* = 8.02 $\times$ 10^–3^) and Na(ClO_4_) (LFC = − 0.69 ± 0.31, *P* = 3.14 $\times$ 10^–2^) treatments was observed. *OpuA*B was also down-regulated in the 0 °C Mg(ClO_4_)_2_ treatment (LFC = − 1.03 ± 0.38, *P* = 1.56 $\times$ 10^–2^).

Evidence for osmotic stress can also be observed in the regulation of cell surface protein synthesis. Methanochondroitin is the primary constituent of the extracellular matrix that clumps *M. barkeri* cells into multicellular aggregates under optimal growth conditions^61^. Glucuronic acid, generated from glucose degradation via UDP-glucose dehydrogenase (*UGDH*), is a major component of methanochondroitin^61,62^. We observed significant down-regulation of *UGDH* under Mg(ClO_4_)_2_ and Na(ClO_4_) conditions at 30 °C (LFC_Mg_ = − 0.55 ± 0.17, *P* = 3 $\times$ 10^–3^; LFC_Na_ = − 0.51 ± 0.17, *P* = 7 $\times$ 10^–3^).

### Down-regulation of sulfur-containing amino acids

**Figure 5:**
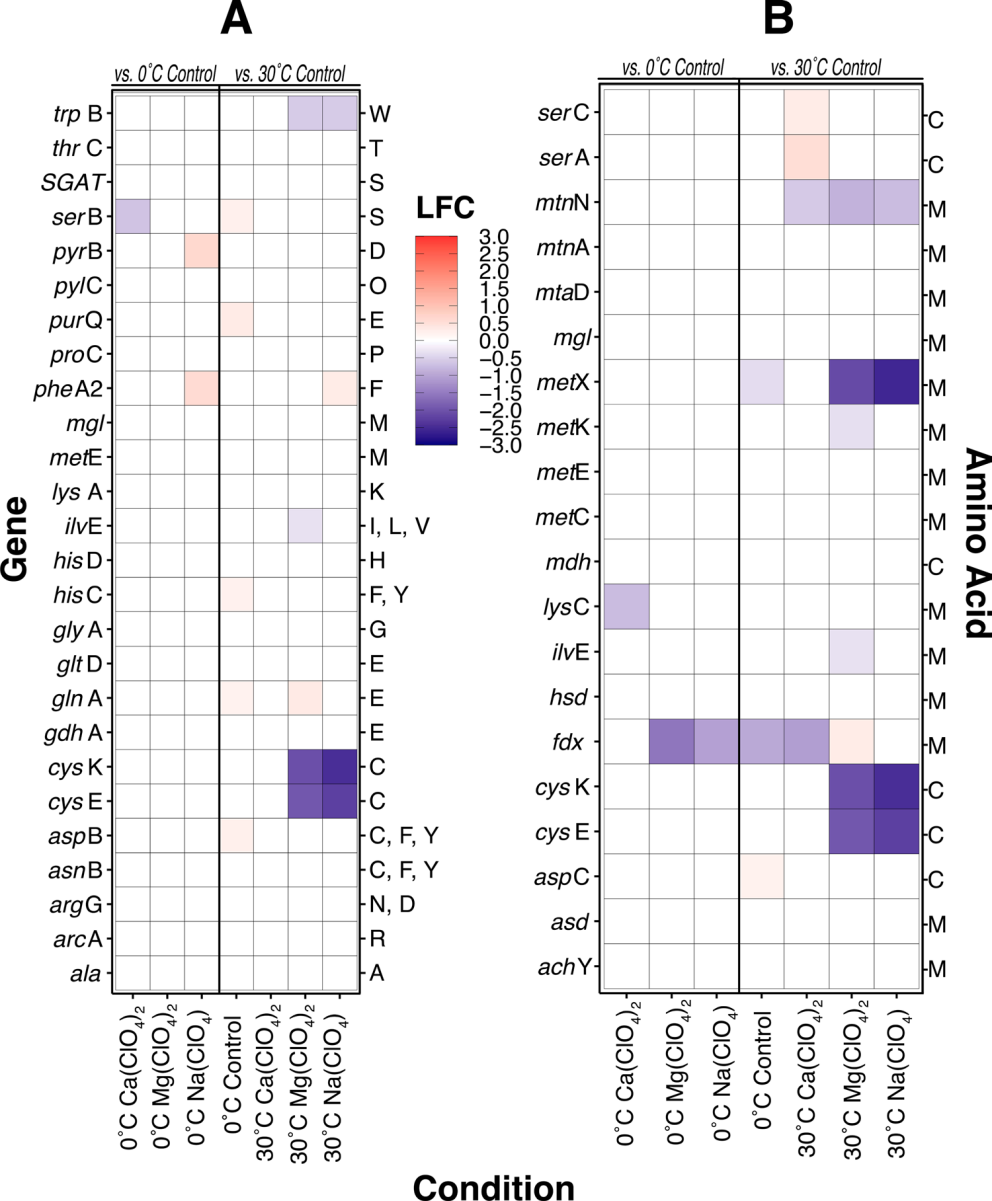
Differential expression (Log_2_-fold change, LFC) of genes involved in (**a**) amino acid synthesis and (**b**) recycling of the sulfur-containing amino acids methionine and cysteine in *M. barkeri* MS. Perchlorate-amended 30 °C and 0 °C perchlorate-free control cultures are relative to 30 °C perchlorate-free control. 0 °C perchlorate-amended cultures are relative to 0 °C perchlorate-free control. Full names of gene and amino acid abbreviations are respectively found in Tables S16 and S17.

In addition to the 20 common amino acids, *M. barkeri* also encodes a 21^st^ residue, pyrrolysine, via the ‘amber’ stop codon UAG^63^. A comparison of the genes encoding amino acid synthesis proteins showed large negative log_2_-fold changes at 30 °C in Mg(ClO_4_)_2_ and Na(ClO_4_)-amended treatments with respect to cysteine-producing proteins cysteine synthase (*cys*K) and serine acetyltransferase (*cys*E) (Fig. 5a). Further examination of complete amino acid metabolic pathways (Figs. S4–S16) revealed that this pattern of substantial gene down-regulation was characteristic of not only cysteine, but also the other sulfur-containing amino acid methionine (Fig. 5b).

## Discussion

In this study we took our inspiration from nearly two decades of research into Martian methane and associated astrobiology analogue studies to investigate how methanogens may survive under challenging conditions analogous of the Martian shallow subsurface, specifically under freezing temperatures and in the presence of chaotropic perchlorate salts. We tracked CH_4_ production and changes in global gene expression in *M. barkeri* MS via RNA-Seq following 28 days’ incubation at 30 °C or 0 °C, with and without 10 mM of dissolved Na-perchlorate and 20 mM of dissolved Mg- or Ca-perchlorate. In accordance with prior findings^25,26^ we observed quantifiable but inhibited methanogenesis in cultures supplemented with perchlorates (Fig. 2). For the first time we present transcriptomic evidence of the underlying biochemistry. Regulatory responses point to unambiguous shifts in amino acid synthesis and recycling, as well as mechanisms to combat increased osmotic stress—not unexpected reactions for an obligate anaerobe exposed to strongly oxidizing and chaotropic perchlorate salts. A surprising regulatory switch was observed in the methanogenesis pathway, with a significant up-regulation of methylamine-utilizing genes, despite these substrates not being present in the media ab initio. Metalloenzymes with molybdenum active sites, including (Mo)-formylmethanofuran dehydrogenase and (Mo)-Nase were among the most significantly up-regulated genes in perchlorate-amended incubations at 30 °C. We contextualize these revelations with a precedent of perchlorate reduction in methanogenic cultures^25,26^, offering new insight into Mo active sites as hydrogenation catalysts for these reactions to proceed.

### Evidence for selection against redox-sensitive amino acids under temperate conditions

Cysteine and methionine are exceptionally sensitive to oxidation by reactive radical species^64^. Both residues have shown strong binding affinities to both perchlorate and perchloric acid, resulting in the oxidation of methionine to methionine sulfoxide and cysteine to sulfonic acid^65^. The susceptibility of cysteine and methionine to react with perchlorate risks degradation of protein structure and function. The extensive and substantial down-regulation of cysteine and methionine metabolic pathways we observed in the presence of Na- and Mg-perchlorates at 30 °C (Fig. 5) suggests a concerted effort by *M. barkeri* to reduce the synthesis and repair of these residues in the presence of these perchlorate species. A dearth of significant differential expression of amino acid-recycling genes (including those specific for cysteine and methionine) in both 0 °C and 0 °C perchlorate treatments demonstrates that freezing conditions are not sufficient to confer a decrease in transcriptional activity. It is clear that the synthesis and processing of essential amino acids in *M. barkeri* must proceed despite the metabolic stresses of frigid temperatures and strong oxidants.

### A role for glycine betaine in cryo- and osmoprotection, and as a potential methylamine precursor

The regulatory patterns of enzyme complexes protecting against osmotic stress like *opuA*ABC coincide with temperature and redox conditions. This operon is up-regulated at 30 °C with Na(ClO_4_) and Mg(ClO_4_)_2_, implying an increased need for cellular machinery to import glycine betaine to combat osmotic stress. In addition to being an osmoprotectant, glycine betaine is also cryoprotective^66^. While perchlorate salts are highly oxidizing, they also depress the freezing point of water. The down-regulation of *opuA*B in Mg(ClO_4_)_2_-amended cultures at 0 °C might suggest a decreased cold shock response in *M. barkeri*, wherein glycine betaine uptake for its cryoprotective properties was not needed under freezing conditions.

The up-regulation of methylamine-specific methanogenesis pathway genes in all three 30 °C perchlorate treatments was unexpected. Methylamines were not present in the media ab initio and ion chromatography mass spectrometry did not reveal the presence of monomethylamine in assessed samples (Table S3)—though if samples had ppb-levels of CH_3_NH_2_, it was diluted below the instrument’s detection limit (see Materials and Methods). Free energy yields of methylamine methanogenesis reactions at 30 °C range from -91 to -143 kJ/mol CH_4_—significantly less than the − 158 kJ/mol CH_4_ of the hydrogenotrophic pathway (Table S4). It has been suggested that glycine betaine may be a potential precursor of trimethylamine (TMA)^67,68^. The observed up-regulation of monomethylamine and dimethylamine permeases *mtm*P and *mtb*P (Fig. 3) imply possible interactions between perchlorates and complex media constituents (e.g., perhaps yeast extract or casitone) that might yield methylaminated species for utilization by *M. barkeri*, though we acknowledge such interpretations are presently speculative as our ion chromatography analyses were not sensitive to the detection of dimethylamine, trimethylamine, or any other methylaminated species.

### Up-regulation of Mo-containing enzymes suggest mechanism for H_2_-dependent perchlorate reduction

Fixed nitrogen was replete under initial incubation conditions (9.3 mM NH_4_Cl plus additional N from complex ingredients like yeast extract and casitone). The significant up-regulation of ammonium transporters (*amt*, Fig. 4), methylamine permeases (*mtm*P and *mtb*P, Fig. 3), and P-II repressors (*nif*I, Fig. 4) in 30 °C perchlorate treatments were consistent with observations of mM-level NH_4_^+^ (Table S3) at the time of sample preservation for RNA-Seq. In *M. barkeri*, NH_4_^+^ concentrations as low as 10 µM have been shown to be inhibitory to N_2_ fixation^53–56^. It is therefore surprising to observe a simultaneous and substantial up-regulation of nitrogenase genes, an overwhelming proportion of which being associated with the molybdenum isoform, (e.g., *nif*DEHK, Fig. 4). Nitrogen fixation is an energetically expensive process, consuming at least 16 (and, by one calculation for methanogens^69^, perhaps more than 50) moles of ATP per mole of N_2_ fixed^70^. The significant increase in expression of the *nif*I repressors in the 30 °C Mg(ClO_4_)_2_ and Na(ClO_4_)-amended treatments suggest a concerted effort to dedicate cellular energy towards signaling the shut off of Mo-Nase activity. What is the purpose of this transcriptional arms race?

Microbial perchlorate reduction has been identified throughout the *Proteobacteria*^35,71,72^, as well as halophilic^73^ and hyperthermophilic *Archaea*^74^. The substrate-specific and Mo-containing perchlorate reductase (*pcr*AB), which reduces perchlorate to chlorite (ClO_2_^-^), and chlorite dismutase (*cld*), which dismutates ClO_2_^-^ into chloride (Cl^-^) and molecular oxygen (O_2_), have been isolated and purified^35^, but homologues of these genes have not been found in the *M. barkeri* genome. However, chlorate- and perchlorate-binding affinities have been documented in structurally similar α- and β-subunits of other Mo-containing DMSO family reductases^35,75,76^. A plethora of inorganic and organic electron donors have also been implicated in microbial perchlorate reduction^35,77–79^ including H_2_, sulphide (S_2_^-^), CH_4_, and yeast extract, which are available constituents in the incubations analyzed in this study. The biochemistry of microbial perchlorate reduction is complex and not yet fully understood. Nonetheless, the following environmental conditions must be met in order for perchlorate reduction to proceed: (1) dissolved O_2_ concentrations must be < 2 mg L^−1^; (2) NO_3_^-^ must be completely consumed; and (3) molybdenum must be bioavailable as molybdate, MoO_4_^2−^, to synthesize Mo cofactors^35,80^ in perchlorate-reducing metalloenzymes. All three conditions were coincidentally satisfied in this study’s incubation conditions.

Perchlorate reduction has been observed in sterile methanogenic media under an 80:20 H_2_:CO_2_ atmosphere^25,26^ and apparently exacerbated in media with the psychrotolerant methanogen *Methanobacterium arcticum* M2, leading to the suggestion that perchlorate could be utilized as a possible electron acceptor in a reversal of methanogenesis – the anaerobic oxidation of methane, AOM^25^ (Eq. 1). To date these interpretations remain unverified. In this study, we similarly observed a significant decrease in perchlorate concentrations in media containing *M. barkeri* – a loss of 17.4% for Mg(ClO_4_)_2_, 26.8% for Ca(ClO_4_)_2_, and 58.9% of Na(ClO_4_) by mass (Table S3). Anion measurements in the perchlorate-amended incubations did yield an increase in total Cl^-^, but only enough to account for 35.5% Mg(ClO_4_)_2_, 5.6% Ca(ClO_4_)_2_, and 44.8% of Na(ClO_4_) apparently reduced (Table S3). We note that due to its co-elution with Cl^-^, we were not able to ascertain the presence of chlorate (ClO_3_^-^). Chlorite (ClO_2_^-^) was not detected in any samples (Table S3). Additional resources and measurements in a future study are necessary to quantify ClO_3_^-^ and other potential intermediates (e.g. chlorinated organics) to fully assess the fate of ClO_4_^-^.

1 $${\text{CH}}_{4} + {\text{ ClO}}_{4}^{ - }\rightleftharpoons {\text{CO}}_{2} + {\text{ Cl}}^{ - } + \, 2{\text{H}}_{2} {\text{O}}$$

Sodium sulphide, Na_2_S, a common reducing agent in anaerobic media, has been previously ruled out as a significant contributor to abiotic perchlorate reduction in methanogenic media (medium MSH in Kral et al.)^26^. Yeast extract is a complex and undefined carbon source, and its inclusion in sterile MSH media has been associated with increased rates of perchlorate reduction compared to a minimal salts medium (medium MM in Kral et al.)^26^. In this study, preliminary measurements of perchlorate concentrations in sterile DSMZ 120a media failed to detect conclusive perchorate reduction (Table S3). More work is necessary to fully explore the composition and role of yeast extract as a potential reducing agent.

The free energy yield of H_2_ oxidation coupled to perchlorate reduction (Eq. 2) is substantial (∆G˚^'^ = − 289 kJ/mol H_2_) but the reaction is kinetically sluggish at ambient conditions and requires a metallic catalyst to overcome its large activation energy^81^.

2 $$4{\text{H}}_{2} + {\text{ ClO}}_{4}^{ - }\rightleftharpoons {\text{Cl}}^{ - } + \, 4{\text{H}}_{2} {\text{O}}$$

Nickel (Ni) has long been known as an excellent hydrogenation catalyst^82^ and is an essential cofactor for hydrogenase activity in methanogens^83^, but an essential role in microbial perchlorate reduction has never been suggested and it is not obvious from our transcriptomic data that perchlorates elicit a universal response by Ni active sites in hydrogenases (e.g. *ech*E, *vht*A, and *frh*α in Fig. 3). Instead, given perchlorate reduction’s known Mo dependency^35,84^ and the additional empirical evidence presented here, we cannot ignore the most parsimonious explanation: the substantial up-regulation of (Mo)-Nase (Fig. 4) and (Mo)-formylmethanofuran dehydrogenase (Fig. 3, Table 1) at 30 °C must be associated with perchlorate reduction. Certainly, a thorough investigation is warranted in order to elucidate finer details of the biochemistry.

### The methylamine methanogenesis pathway as a mechanism for energy conservation

In methylamine methanogenesis, H_2_ is recycled by the partial reversal of the methanogenesis pathway, generated via the oxidation of F_420_H_2_ by *frh*α and the oxidation of ferredoxin by *ech*F (green arrows in Figs. 3). H_2_ generated by the oxidation of F_420_H_2_ and ferredoxin diffuses across the membrane to be oxidized by *vht*A in an energy conserving scheme to recycle methanophenazine (MP)^85–88^. Ferredoxin oxidation by *ech*F also results in the translocation of 2H^+^ through *ech*, contributing to the production of a proton gradient (high outside the cell)^88^. Evidence for the generation of this proton gradient might be indicated in our incubations based on drops in pH (up to 0.77 pH units) (Table S5). Based on up-regulation of the genes for methylamine methanogenesis, we attribute the differential expression of *ech*, *vht*, and *frh* hydrogenases (Fig. 3) and ferredoxin (Tables S10, S12, S14) as putative sources of a proton motive force in the 30 °C Ca(ClO_4_)_2_-supplemented incubations.

**Figure 6:**
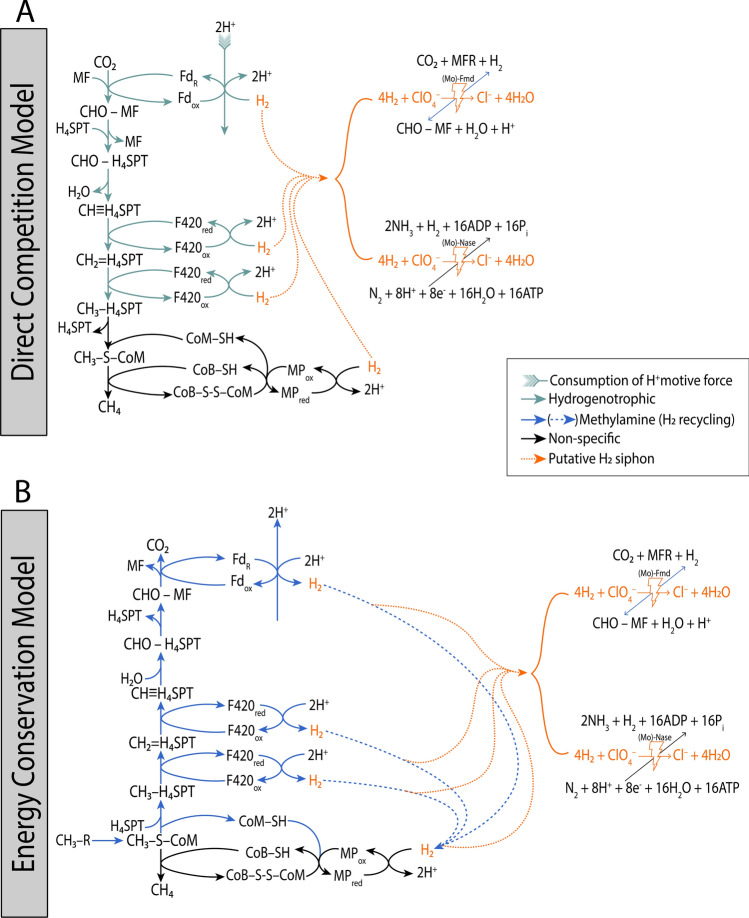
Proposed models of Mo- and H_2_-dependent perchlorate reduction occurring (**a**) in direct competition with hydrogenotrophic methanogenesis, and (**b**) via the energy-conserving partial reversal of methylamine-based methanogenesis. Abbreviations: Non-specific, common to multiple pathways; MF, methanofuran; CHO-MF, formyl-methanofuran; H_4_SPT, tetrahydrosarcinapterin; CHO-H_4_SPT, formyl-tetrahydrosarcinapterin; CH≡H_4_SPT, methenyl-tetrahydrosarcinapterin; CH_2_ = H_4_SPT, methylene-tetrahydrosarcinapterin; CH_3_-H_4_SPT, methyl-tetrahydrosarcinapterin; CoM-SH, coenzyme M; CoB-SH, coenzyme B; CoB-S–S-CoM, CoB-CoM heterodisulphide; CH_3_-S-CoM, methyl-coenzyme M; CH_3_-R, methylamine; MP, methanophenazine; Fd, ferredoxin; F420, coenzyme F420; (Mo)-Fmd, (Mo)-formylmethanofuran dehydrogenase; (Mo)-Nase, (Mo)-nitrogenase.

We theorize that the thermodynamic spontaneity of H_2_-dependent perchlorate reduction (in the presence of a Mo catalyst) results in it outcompeting the endergonic first step of hydrogenotrophic methanogenesis (Eq. 3; ∆G˚^'^ =  + 16 kJ/mol H_2_)^89^ (Fig. 6a).

Likewise, we imagine H_2_ intermediates generated during energy conserving steps of methylamine methanogenesis are siphoned off to reduce perchlorate in lieu of reducing MP via *vht* (∆G˚^'^ = − 289 kJ/mol H_2_ for perchlorate reduction^90^ vs. ∆G˚^'^ = − 50 kJ/mol H_2_ for MP reduction^91,92^) (Fig. 6b). Such interpretations warrant further investigation, but do fit within previously reported narratives of observed decreases in CH_4_ production in perchlorate-supplemented methanogenic cultures^25,26^.

3 $$\begin{aligned} & {\text{CO}}_{2} + {^{\dag }\text{MFR-NH}}_{3}^{+} + {\text{ H}}_{2} \rightleftharpoons {^{\dag \dag }\text{CHO-NH-MFR}}+ {\text{ H}}_{2} {\text{O }} + {\text{ H}}^{ + }  \\  & {}^{\dag } {\text{Methanofuran}}  \\ & {}^{\dag \dag } {\text{Formyl - methanofuran}}  \\ \end{aligned}$$

### Do all perchlorates trigger universal regulatory responses?

Calcium-, magnesium- and sodium perchlorate were investigated in this study as they have been detected in active Recurring Slope Lineae (RSL) on Mars^43^. Until now, studies investigating methanogen survival in high concentrations of perchlorate salts have been unable to ascertain the specific metabolic responses behind observed decreases in CH_4_ production, and whether these metabolic responses are universal regardless of the perchlorate’s constituent cation. Our work demonstrates that, for *M. barkeri* MS transcriptomes assembled after 28 days of incubation, methanogenesis regulatory patterns are consistent across all three perchlorate conditions with methylamine gene utilization and a handful of (Mo)-*fmd* subunits at 30 °C (Fig. 3). Otherwise, global expression patterns in Ca(ClO_4_)_2_ treatments were somewhat distinct from Na(ClO_4_) and Mg(ClO_4_)_2_ conditions, which, on the whole, exhibited similar transcriptomic responses at both temperatures (Figs. 4, 5; Tables 1, 2). Transcriptomes from additional time points can help clarify whether these discrepancies in expression are reflective of distinct metabolic responses to Ca-perchlorate versus Mg- and Na-perchlorate, or simply different stages of metabolism following prolonged exposure to perchlorates in general. We infer the latter is more likely given the novel and ubiquitous response of genes in the methylamine methanogenesis pathway.

### Implications for astrobiology and Martian methane

These findings better constrain our growing understanding of how microbial life responds to prolonged exposure under extreme conditions characterized by strong oxidants, freezing temperatures, and osmotic stress—e.g., a Mars Special Region^93^ potentially conducive to microbial habitability. Notably, our study shows that major metabolic disruption by perchlorates at 30 °C is not reflected to the same degree at 0 °C, which is more appropriately representative of the conditions at Mars Special Regions^93^. This finding offers new perspectives to contextualize observations of diffuse Martian CH_4_ emissions, particularly in situ measurements made by the Curiosity rover^5,6^. This work offers an in-depth glimpse into the remarkable adaptability of methanogens to survive under oxidative stresses that mimic the challenging conditions of the Martian subsurface. The inferences made from this study provide many exciting opportunities for further research to better understand methanogen ecophysiology under extreme stress that may offer terrestrial insight into the enigmatic Martian methane story.

## Materials and methods

### Materials and culture conditions

*Methanosarcina barkeri* wild-type strain MS (ATCC 51582) was grown hydrogenotrophically (15 mL of 80:20 H_2_:CO_2_ headspace pressurized to 1.5 bar) in anaerobic balch tubes (Chemglass Life Sciences LLC, Vineland, NJ USA) containing 9 mL DSMZ medium 120a, 10 mg/L EDTA (pH 7.0 to 7.2)^94^, supplemented with trace element solution SL-10^95^ and vitamin supplement MD-VS (ATCC, Manassas, VA USA). Media assaying for perchlorate tolerance were augmented to final concentrations of 10 mM NaClO_4_ and 20 mM Mg(ClO_4_)_2_ or Ca(ClO_4_)_2_. Balch tubes were inoculated with 1 mL cell suspensions containing ~ 4 × 10^7^ cells and incubated in batches of 9 biological replicates plus one autoclave-sterilized media blank for 28 days (Fig. S17). For each experimental treatment (perchlorate-free control, NaClO_4_, Mg(ClO_4_)_2_, or Ca(ClO_4_)_2_), optical density measurements (OD) and CH_4_ production were monitored weekly in parallel experiments incubated at 30 °C or 0 °C (Fig. 2).

OD measurements were performed spectrophotometrically at 600 nm using a Genesys 30 Visible Spectrometer (Thermo-Scientific Corp., Madison, WI USA). At the end of the incubation experiment, direct cell counts were performed on enrichment aliquots fluorescently stained with Acridine Orange (AO) dye (catalogue #318,337, Sigma-Aldrich Chemical, Co., Milwaukee, WI USA) (50 µM final concentration) following an established procedure^96^. Stained cells were enumerated in a Spotlite hemocytometer counting chamber (McGaw Park, IL USA) with a Zeiss Axioskope 40 epifluorescence microscope (ZEISS Microscopy, Jena, Germany) at 600 × magnification using an AO filter cube set (excitation 470/20 nm; emission > 510 nm).

Headspace gas samples were collected via gas-tight syringes (catalogue #24886, Restek U.S., Bellefonte, PA USA) to measure CH_4_ using a Trace 1310 gas chromatograph equipped with a flame-ionizing detector (GC-FID) (ThermoFisher Scientific, Waltham, MA USA). H_2_, O_2_, and N_2_ was were measured using a Peak Performer 1 series gas chromatograph coupled to a thermal conductivity detector (GC-TCD; Peak Laboratories, Mountain View, CA USA) and CO_2_ and CH_4_ were measured using an FID coupled to a methanizer (GC-FID; Peak Laboratories, Mountain View, CA USA). To avoid injecting atmospheric O_2_ and residual carrier-over of trace gases within the syringes between samples, all syringes were flushed three times with ultra-high purity argon gas between samples.

Single experimental replicates from each condition were reserved for measurement of anions (ClO_4_^−^, ClO_2_^−^, F^−^, Cl^−^, SO_4_^2−^, NO_2_^−^, NO_3_^−^, HPO_4_^2−^), major cations (Na^+^, Mg^2+^, Ca^2+^, K^+^), ammonium (NH_4_^+^), and monomethylamine (CH_3_NH_2_). Media was diluted 500 × in milliQ H_2_O and compositional analysis was performed by ion chromatography on a Dionex ICS-5000 + Capillary HPIC system (ThermoFisher Scientific, Waltham, MA USA). Perchlorate (ClO_4_^−^) and chlorite (ClO_2_^−^) were separated from other anions using a Dionex IonPac AS16 column (ThermoFisher Scientific, Waltham, MA USA). We note that chlorate (ClO_3_^-^) coeluted with Cl^-^ and could not be distinguished. TraceCERT perchlorate standard (catalogue # 76462, Sigma-Aldrich Chemical, Co., Milwaukee, WI USA) and Dionex Combined Seven Anion II (catalogue # 057590, ThermoFisher Scientific, Waltham, MA USA) were used for standardization. Major cations, NH_4_^+^, and CH_3_NH_2_ were measured by diluting the media 100 × in milliQ H_2_O on the same ion chromatograph fitted with a CS16 column (ThermoFisher Scientific, Waltham, MA USA), and was standardized by the Dionex Combined Six Cation solution (catalogue #046070, ThermoFisher Scientific, Waltham, MA USA) and monomethylamine solution 40% by wt. in H_2_O (Sigma-Aldrich catalogue # 426466).

Compositional analyses of cations, anions, and headspace gas is available for reference in Table S3.

### RNA isolation and purification

After 28 days’ incubation, culture and blank volume contents were briefly vortexed and aseptically transferred into sterile 15 mL Falcon tubes (Corning Inc., Corning, NY USA) at incubation temperature inside a Coy anaerobic chamber (Coy Laboratory Products Inc., Grass Lake, MI USA). Tubes were centrifuged at 3,000 × g for 1 min using an IEC Centra CL2 centrifuge (Thermo Electron Company, Milford, MA USA) to pellet cells. Media was poured off for pH and electrical conductivity measurements, leaving behind 1 mL. To ensure quantifiable RNA yields downstream, the nine biological replicates from each condition were consolidated into three sets of three samples each for extraction (3 mL pelleted cell suspension/tube). RNAlater solution (ThermoFisher Scientific, Waltham, MA USA) was added to a final volume of ~ 12 mL. Samples were left to equilibrate at incubation temperature for 3 h, transferred to 4 °C for 24 h, and then stored at -80 °C until overnight shipment on dry ice to Princeton University for RNA extraction.

Samples preserved at -80 °C in RNAlater were thawed on ice in a sealed container before contents were transferred to 50 mL Falcon® tubes. An equal volume (12 mL) of nuclease-free water (Qiagen, Hilden, Germany) was added to RNAlater-preserved samples, briefly vortexed, and centrifuged at 5000 gx for 10 min using a Sorvall Legend XI centrifuge (ThermoFisher Scientific, Waltham, MA USA). The supernatant was subsequently discarded, and RNA was extracted following a modified protocol from a Zymo Quick-RNA Miniprep Plus Kit (Zymo Research, Irvine, CA USA). RNA lysis buffer and nuclease-free water were added to each sample in a 5:1 ratio, and sterile 0.7 mm garnet bashing beads (Qiagen, Hilden, Germany) were added to facilitate mechanical lysis during subsequent vortexing. Samples were then vortexed for 1 min and centrifuged at 10,000 × g at 4 °C for 1 min using an Eppendorf 5810R (Eppendorf, Hamburg, Germany) to pellet cell debris. The supernatant containing total nucleic acids was transferred to a yellow Spin-Away column (Zymo Research, Irvine, CA USA) fitted in a 2 ml collection tube. Samples were centrifuged at 10,000 × g for 1 min using an AccuSpin Micro 17 (ThermoFisher Scientific, Waltham, MA USA) to separate out genomic DNA. Following centrifugation, the Spin-Away filter was discarded and the filtrate was collected from the column for RNA purification.

Total nucleic acids were then precipitated by adding 1 mL of chilled absolute ethanol. Pellets were mixed by pipetting and then transferred to green Zymo-Spin IIICG column filters fitted in clean collection tubes. Samples were centrifuged at 10,000 × g for 30 s to collect precipitated nucleic acids on the column, subsequently treated with 400 µL RNA wash buffer, and centrifuged for 30 s at 10,000 gx. The wash buffer was discarded and columns were treated with 80 µL DNAse I reaction mixture (per 80 µL: 5 µL DNAse 1 [1 U/µL], 8 µL 10X DNAse I reaction buffer [Zymo Research, Irvine, CA USA], 3 µL nuclease-free water, 64 µL RNA wash buffer [Zymo Research, Irvine, CA USA]) to degrade trace genomic DNA and left to incubate on ice in the dark for 15 min. DNAse-treated samples were then centrifuged at 10,000 × g for 30 s. The reaction buffers were discarded, column filters were washed three times with 400, 700, and 400 µl of RNA Prep Buffer (Zymo Research, Irvine, CA USA), centrifuging twice for 30 s at 10,000 × g and at 16,000 × g for 2 min for the final wash. Total RNA was then eluted into sterile, nuclease-free PCR tubes on ice using nuclease-free water pre-heated to 95﻿ °C. Total RNA was quantified using a Qubit hs RNA assay kit coupled to a Qubit 2.0 analyzer (ThermoFisher Scientific, Waltham, MA USA) following the manufacturer’s protocol. RNA quality was subsequently assessed using a 2100 Bioanalyzer (Agilent Technologies Inc., Santa Clara, CA USA). RNA samples were kept at -20 °C until library preparation and sequencing.

All RNA extraction steps were performed using nuclease-free pipette tips (Corning Inc., Corning, NY USA) in a UV-sterilized laminar flow hood. All surfaces, pipettes, and gloves were wiped down at each step with RNaseZap solution (ThermoFisher Scientific, Waltham, MA USA) to minimize potential RNase contamination. Parallel extraction blanks of extraction kit reagents and blank growth media co-incubated with *M. barkeri* enrichment cultures were performed to ensure cleanliness of the extraction procedure and sterility of uninoculated growth media.

### RNA library prep and RNA-Seq

Ribosomal RNA was depleted from total RNA using a Ribo-Zero Bacterial rRNA Removal Kit (Illumina, Inc., San Diego, CA USA) following the manufacturer’s instructions and using the provided universal probe sequence. One-directional library prep was performed for each treatment and its constituent 3 sequencing replicates using the Nextera DNA Flex Library Prep Kit (Illumina, Inc., San Diego, CA USA) on the automated Apollo 324 system (Takara Bio USA, Inc., Mountain View, CA, USA). RNA-Seq was carried out for 318 cycles on two lanes of a NovaSeq SP Flowcell (Illumina, Inc., San Diego, CA USA) (1 × 150 bp) at the Princeton University genomics core facility.

### Annotation and comparative transcriptomics

Quality filtering of single-end reads was performed using fastp v.0.12.6^97^, removing reads < 50 nt, containing > 1 Ns, Phred quality scores < 30, and sample barcode sequences. Using Bowtie2 v.2.3.2^98^, retained, quality-filtered reads from each experiment were mapped to coding sequence (CDS) regions subset from the complete *M. barkeri* MS reference genome obtained from NCBI GenBank (NZ_CP009528.1). CDS-mapped reads were then sorted, indexed, and processed for extraction from the sequenced transcriptome using Samtools v.1.5^99^ and BEDTools v.2.17.0^100^. Gene annotation was performed using NCBI BLASTn v.2.2.29 + ^101^ against a reference *M. barkeri* MS CDS assembly database generated using option -makeblastdb. Protein assignment was determined as the entry with the greatest sequence identity alignment with the query sequence, the lowest E-value, and largest bit score. Differential expression analysis of investigated treatments relative to the 30 °C and 0 °C perchlorate-free controls, within-group (i.e. biological replicate) variance estimation, and FPM were performed using DESeq2^102^. Metabolic pathway involvement of identified genes was determined by referencing the Kyoto Encyclopedia of Genes and Genomes (KEGG)^103^.

## Supplementary Information

Supplementary Information 1.

Supplementary Information 2.

Supplementary Information 3.

Supplementary Information 4.

## Data Availability

RNA-Seq data is available at NCBI GenBank under accession number PRJNA635445 (Sequence Read Archive SRP265010). The source code of all transcriptomic analyses presented in this manuscript are available upon request.
