# Supplementary material for: Transcriptional response to prolonged perchlorate exposure in the methanogen Methanosarcina barkeri and implications for Martian habitability
Source: Sci Rep. 2021 Jun 11;11:12336. doi: 10.1038/s41598-021-91882-0 (PMC8196204; doi:10.1038/s41598-021-91882-0)
Supplement: Supplementary file 1 — Supplementary Information 1. [file 41598_2021_91882_MOESM1_ESM.docx]

SUPPLEMENTARY INFORMATION

**Transcriptional response to prolonged perchlorate exposure in the methanogen *Methanosarcina barkeri* and implications for Martian habitability**

**Authors**: Rachel L. Harris^*^, Andrew C. Schuerger, Wei Wang, Yuri Tamama, Zachary K. Garvin, and Tullis C. Onstott

This document contains:

Figures S1 – S17

Tables S1, S4 – S5, S13 – S17

Tables S2 – S3 and S6 – S12 can be found as additional .xlsx files

**SUPPLEMENTARY FIGURES**

|  |
| --- |
| **Figure S1**. Average ± SD transcript fragment counts per million mapped reads (FPM) at 30˚C. (n = 3 libraries per condition). Open reading frame number indicates position in genome (where ORF = 1 is the origin of replication). ORFs reference gene products found in Table S2. |

|  |
| --- |
| **Figure S2**. Average ± SD transcript fragment counts per million mapped reads (FPM) at 0˚C. (n = 3 libraries per condition). Open reading frame number indicates position in genome (where ORF = 1 is the origin of replication). ORFs reference gene products found in Table S2. |

| 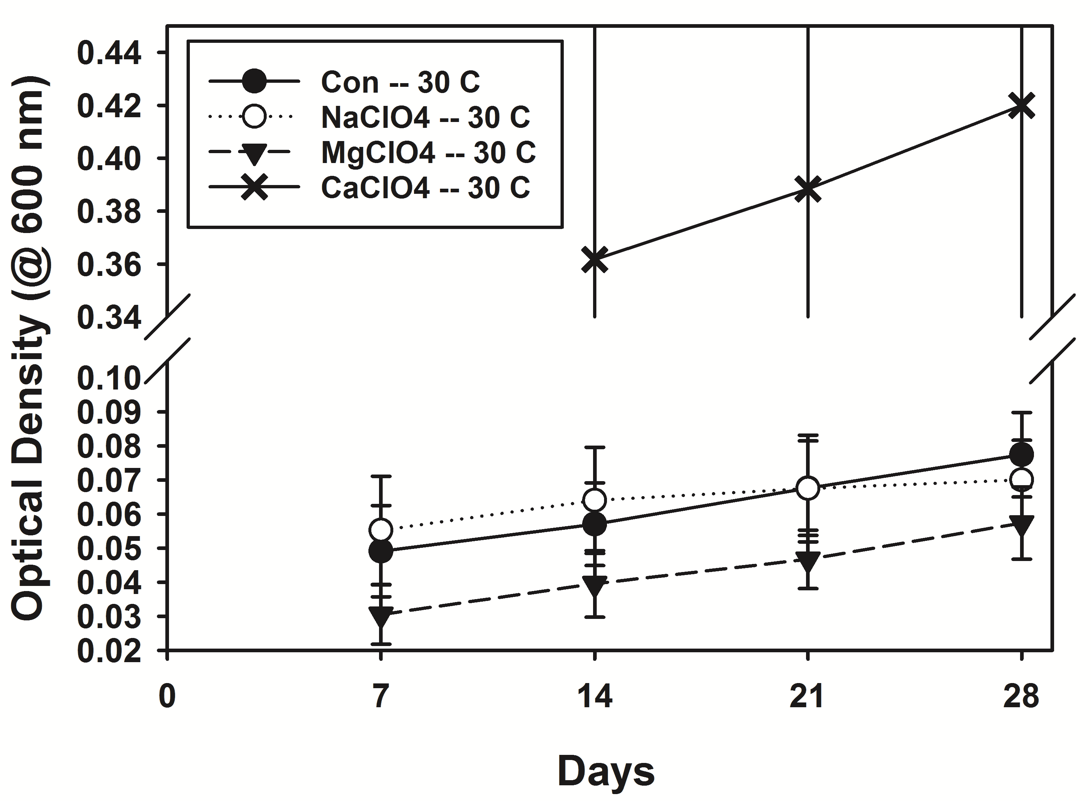**A** |
| --- |
| 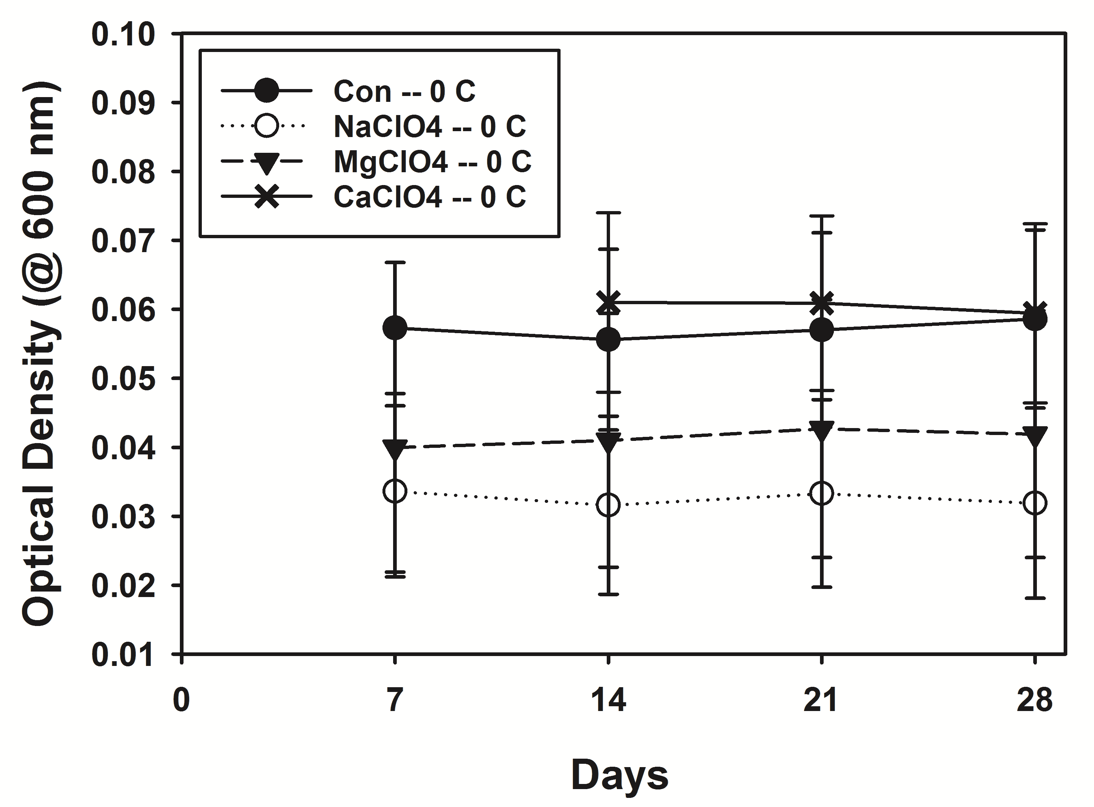**B** |
| **Figure S3.** Weekly OD_600_ ± SD of *Methanosarcina barkeri* grown at 30˚C **(A)** and 0˚C **(B)**. |
| **** |
| **Figure S4**. Differential expression (Log_2_-fold change, LFC) of genes involved in alanine, aspartate, and glutamate metabolisms in *M. barkeri*. Perchlorate-amended 30˚C and 0˚C perchlorate-free control cultures are relative to 30˚C perchlorate-free control. 0˚C perchlorate-amended cultures are relative to 0˚C perchlorate-free control. Significant differential expression was identified via Wald test (*P* < 0.05). Gene abbreviations: *puu*E, 4-aminobutyrate aminotransferase; *pur*B, Adenylosuccinate lyase; *pur*A, Adenylosuccinate synthase; *pur*F, Amidophosphoribosyltransferase; *arg*H, Arginosuccinate lyase; *arg*G, Argininosuccinate synthase; *asn*B, Asparagine synthetase; *asp*C, Aspartate aminotransferase; *pyr*I, Aspartate carbamoyltransferase regulatory subunit; *asp*B, Biosynthetic aromatic amino acid aminotransferase alpha; *car*B, Carbamoyl-phosphate synthase large chain; *car*A, Carbamoyl-phosphate synthase small subunit; *glm*S, Glucosamine—fructose-6-phosphate aminotransferase; *gad*AB, Glutamate decarboxylase; *gdh*A, Glutamate dehydrogenase; *glt*D, Glutamate synthase [NADPH] large chain; *gln*A, Glutamine synthetase type I, *ala*, Ornithine cyclodeaminase; *pur*M, Phosphoribosylformylglycinamidine cyclo-ligase; *gab*D, Succinate-semialdehyde dehydrogenase. |

| **** |
| --- |
| **Figure S5**. Differential expression (Log_2_-fold change, LFC) of genes involved in arginine biosynthesis in *M. barkeri*. Perchlorate-amended 30˚C and 0˚C perchlorate-free control cultures are relative to 30˚C perchlorate-free control. 0˚C perchlorate-amended cultures are relative to 0˚C perchlorate-free control. Significant differential expression was identified via Wald test (*P* < 0.05). Gene abbreviations: *arg*B, Acetylglutamate kinase; *arg*D, Acetylornithine aminotransferase; *arc*A, Arginine deaminase; *arg*H, Arginosuccinate lyase; *arg*G, Arginosuccinate synthase; *asp*C, Aspartate aminotransferase; *asp*B, Biosynthetic aromatic amino acid aminotransferase alpha; *arg*A, GCN5-related N-acetyltransferase; *gdh*A, Glutamate dehydrogenase; *gln*A, Glutamine synthetase type I; *arg*J, Glutamate N-acetyltransferase; *arg*C, N-acetyl-gamma-glutamyl-phosphate reductase; *arg*F, Ornithine carbamoyltransferase; *pur*Q, Phosphoribosylformylglycinamidine synthase, glutamine amidotransferase subunit. |

| **** |
| --- |
| **Figure S6**. Differential expression (Log_2_-fold change, LFC) of genes involved in arginine and proline metabolisms in *M. barkeri*. Perchlorate-amended 30˚C and 0˚C perchlorate-free control cultures are relative to 30˚C perchlorate-free control. 0˚C perchlorate-amended cultures are relative to 0˚C perchlorate-free control. Significant differential expression was identified via Wald test (*P* < 0.05). Gene abbreviations: *spe*B, Agmatinase; *asp*C, Aspartate aminotransferase; *asp*B, Biosynthetic aromatic amino acid aminotransferase alpha; *FCY*1, Cytosine deaminase; *pro*A, Gamma-glutamyl phosphate reductase; *pro*B, Glutamate 5-kinase; *pro*C, Pyrroline 5-carboxylate reductase; *pda*D, Pyruvoyl-dependent arginine decarboxylase. |

| **** |
| --- |
| **Figure S7**. Differential expression (Log_2_-fold change, LFC) of genes involved in glycine, serine, and threonine metabolisms in *M. barkeri*. Perchlorate-amended 30˚C and 0˚C perchlorate-free control cultures are relative to 30˚C perchlorate-free control. 0˚C perchlorate-amended cultures are relative to 0˚C perchlorate-free control. Significant differential expression was identified via Wald test (*P* < 0.05). Gene abbreviations: *asd*, Aspartate-semialdehyde dehydrogenase; *lys*C, Aspartokinase; *pss*, CDP-diaglycerol-serine O-phosphatidyltransferase; *ser*A, D-3-phosphoglycerate dehydrogenase; *hsd*, Homoserine dehydrogenase; *gpm*A, Phosphoglycerate mutase; *ser*C, Phosphoserine aminotransferase; *ser*B, Phosphoserine phosphatase, *gly*A, Serine hydroxymethyltransferase/L-threonine aldolase; *SGAT*, Serine--glyoxylate aminotransferase; *thr*C, Threonine synthase; *trp*B, Tryptophan synthase beta chain. |

| **** |
| --- |
| **Figure S8**. Differential expression (Log_2_-fold change, LFC) of genes involved in histidine metabolism in *M. barkeri*. Perchlorate-amended 30˚C and 0˚C perchlorate-free control cultures are relative to 30˚C perchlorate-free control. 0˚C perchlorate-amended cultures are relative to 0˚C perchlorate-free control. Significant differential expression was identified via Wald test (*P* < 0.05). Gene abbreviations: *asp*C, Aspartate aminotransferase; *his*G, ATP phosphoribosyltransferase; *asp*B, Biosynthetic aromatic amino acid aminotransferase alpha; *his*D, Histidinol dehydrogenase; *his*B, Imidazoleglycerol-phosphate dehydratase; *his*E, Phosphoribosyl-ATP pyrophosphatase; *his*A, Phosphoribosylformimino-5-aminoimidazole carboxamide ribotide isomerase. |

| **** |
| --- |
| **Figure S9**. Differential expression (Log_2_-fold change, LFC) of genes involved in lysine and pyrrolysine biosynthesis in *M. barkeri*. Perchlorate-amended 30˚C and 0˚C perchlorate-free control cultures are relative to 30˚C perchlorate-free control. 0˚C perchlorate-amended cultures are relative to 0˚C perchlorate-free control. Significant differential expression was identified via Wald test (*P* < 0.05). Gene abbreviations: *arg*D, Acetylornithine aminotransferase; *asd*, Aspartate-semialdehyde dehydrogenase; *lys*C, Aspartokinase; *aks*A, Coenzyme B synthesis from 2-oxoglutarate: steps 1, 6, and 10; *aks*D, Coenzyme B synthesis from 2-oxoglutarate: steps 4, 7, 8, 11, and 12 (large subunit); *aks*E, Coeznyme B synthesis from 2-oxoglutarate: steps 4, 7, 8, 11, and 12 (small subunit); *lys*A, Diaminopimelate decarboxylase; *dap*B, Dihydrodipicolinate reductase; *dap*A, Dihydrodipicolinate synthase; *hom*; Homoserine dehydrogenase; *dap*L, LL-diaminopimelate aminotransferase; *pyl*B, Proline 2-methylase for pyrrolysine biosynthesis; *pyl*D, Proline reductase for pyrrolysine biosynthesis; *pyl*C, Pyrrolysine synthetase. |

| **** |
| --- |
| **Figure S10**. Differential expression (Log_2_-fold change, LFC) of genes involved in lysine degradation in *M. barkeri*. Perchlorate-amended 30˚C and 0˚C perchlorate-free control cultures are relative to 30˚C perchlorate-free control. 0˚C perchlorate-amended cultures are relative to 0˚C perchlorate-free control. Significant differential expression was identified via Wald test (*P* < 0.05). Gene abbreviations: *kam*A, Lysine 2,3-aminomutase; *gab*D, Succinate-semialdehyde dehydrogenase [NAD]. |

| **** |
| --- |
| **Figure S11**. Differential expression (Log_2_-fold change, LFC) of genes involved in phenylalanine metabolism in *M. barkeri*. Perchlorate-amended 30˚C and 0˚C perchlorate-free control cultures are relative to 30˚C perchlorate-free control. 0˚C perchlorate-amended cultures are relative to 0˚C perchlorate-free control. Significant differential expression was identified via Wald test (*P* < 0.05). Gene abbreviations: *asp*C, Aspartate aminotransferase; *asp*B, Biosynthetic aromatic amino acid aminotransferase alpha; *kat*G, Catalase; *paa*I, Phenylacetic acid degradation protein; *paa*K, Phenylacetate-CoA ligase. |

| **** |
| --- |
| **Figure S12**. Differential expression (Log_2_-fold change, LFC) of genes involved in phenylalanine, tyrosine, and tryptophan metabolism in *M. barkeri*. Perchlorate-amended 30˚C and 0˚C perchlorate-free control cultures are relative to 30˚C perchlorate-free control. 0˚C perchlorate-amended cultures are relative to 0˚C perchlorate-free control. Significant differential expression was identified via Wald test (*P* < 0.05). Gene abbreviations: *adh*, 2-amino-3,7 dideoxy-D-threo-hept-6-ulosonate synthase; *aro*D, 3-dehydroquinate dehydratase I; *aro*B, 3,7-dideoxy-D-threo-hepto-2,6-diulosonate synthase; *aro*A, 5-Enolpyruvylshikimate-3-phosphate synthase; *trp*D, Anthranilate phosphoribosyltransferase; *trp*E, Anthranilate synthase, aminase component; *asp*C, Aspartate aminotransferase; *asp*B, Biosynthetic aromatic amino acid aminotransferase alpha; *phe*A1, Chorismate mutase I; *aro*C, Chorismate synthase; *ald*A, Fructose-bisphosphate aldolase; *trp*C, Indole-3-glycerol phosphate synthase; *trp*F, Phosphoribosylanthranilate isomerase; *try*A2, Prephenate and/or arogenate dehydrogenase; *phe*A2, Prephenate dehydratase; *aro*E, Shikimate 5-dehydrogenase I alpha; *aro*K, Shikimate kinase II; *trp*A, Tryptophan synthase alpha chain; *trp*B, Tryptophan synthase beta chain. |

| **** |
| --- |
| **Figure S13**. Differential expression (Log_2_-fold change, LFC) of genes involved in tryptophan metabolism in *M. barkeri*. Perchlorate-amended 30˚C and 0˚C perchlorate-free control cultures are relative to 30˚C perchlorate-free control. 0˚C perchlorate-amended cultures are relative to 0˚C perchlorate-free control. Significant differential expression was identified via Wald test (*P* < 0.05). Gene abbreviations: *ato*B, Beta-ketoacyl synthase/thiolase; *kat*G, Catalase; *kyn*B, Metal-dependent hydrolase; *ipd*C, Pyruvate decarboxylase. |

| **** |
| --- |
| **Figure S14**. Differential expression (Log_2_-fold change, LFC) of genes involved in tyrosine metabolism in *M. barkeri*. Perchlorate-amended 30˚C and 0˚C perchlorate-free control cultures are relative to 30˚C perchlorate-free control. 0˚C perchlorate-amended cultures are relative to 0˚C perchlorate-free control. Significant differential expression was identified via Wald test (*P* < 0.05). Gene abbreviations: *yia*Y, Alcohol dehydrogenase; *asp*C, Aspartate aminotransferase; *asp*B, Biosynthetic aromatic amino acid aminotransferase alpha; *mfn*A, L-tyrosine decarboxylase; *gab*D, Succinate-semialdehyde dehydrogenase [NAD]. |

| **** |
| --- |
| **Figure S15**. Differential expression (Log_2_-fold change, LFC) of genes involved in valine, leucine, and isoleucine biosynthesis in *M. barkeri*. Perchlorate-amended 30˚C and 0˚C perchlorate-free control cultures are relative to 30˚C perchlorate-free control. 0˚C perchlorate-amended cultures are relative to 0˚C perchlorate-free control. Significant differential expression was identified via Wald test (*P* < 0.05). Gene abbreviations: *cim*A, (R)-citramalate synthase; *leu*A, 2-isopropylmalate synthase; *leu*C, 3-isopropylmalate dehydratase; *ilv*I, Acetolactate synthase large subunit; *ilv*G, Acetolactate synthase small subunit; i*lv*E, Branched-chain amino acid aminotransferase alpha; *ilv*D, Dihydroxy-acid dehydratase; *ilv*C, Ketol-acid reductoisomerase. |

| **** |
| --- |
| **Figure S16**. Differential expression (Log_2_-fold change, LFC) of genes involved in valine, leucine, and isoleucine degradation in *M. barkeri*. Perchlorate-amended 30˚C and 0˚C perchlorate-free control cultures are relative to 30˚C perchlorate-free control. 0˚C perchlorate-amended cultures are relative to 0˚C perchlorate-free control. Significant differential expression was identified via Wald test (*P* < 0.05). Gene abbreviations: *frn*L, Beta-ketoacyl synthase/thiolase; *ilv*E, Branched-chain amino acid aminotransferase; *hmg*S, Hydroxymethylglutaryl-CoA synthase; *vor*A, Ketoisovalerate oxidoreductase. |

|  |
| --- |
| **Figure S17.** Experimental growth conditions investigating temperature and perchlorate effects on *M. barkeri* MS. |

**SUPPLEMENTARY TABLES**

| **Table S1.** RNA-Seq mapping results of quality-filtered reads, reported as average % mapping (± SD) (n = 3 libraries per condition). Controls are perchlorate-free incubations. Abbreviations: CDS, coding sequence. | | | | |
| --- | --- | --- | --- | --- |
| Temperature (˚C) | Condition | % mapped to reference genome | % mapped rRNA genes | % mapped CDS regions |
| 30 | Control | 95.47  (2.28) | 90.74 (3.44) | 1.14  (0.22) |
| 30 | Na(ClO_4_) | 97.29  (0.79) | 93.68  (1.80) | 1.39  (0.58) |
| 30 | Mg(ClO_4_)_2_ | 97.55  (0.74) | 89.81  (1.13) | 2.94  (0.11) |
| 30 | Ca(ClO_4_)_2_ | 97.05  (1.50) | 94.27  (1.50) | 0.91  (0.23) |
| 0 | Control | 97.15  (0.93) | 91.81  (1.29) | 1.71  (0.42) |
| 0 | Na(ClO_4_) | 97.18  (0.59) | 92.42  (1.18) | 1.67  (0.04) |
| 0 | Mg(ClO_4_)_2_ | 97.58  (0.76) | 93.36  (1.84) | 1.07  (0.16) |
| 0 | Ca(ClO_4_)_2_ | 96.85  (0.96) | 93.19  (0.94) | 1.55  (0.24) |

**Tables S2 – S3** can be found as additional .xlsx files.

| **Table S4**. Gibbs free energy change (∆G˚_Rxn_) of methanogenesis net reactions at 30˚C and 0˚C. Values are presented in kJ/mol CH_4_. | | |
| --- | --- | --- |
| **Reaction** | **30˚C** | **0˚C** |
| $\text{H}^{\text{+}}\text{+}\text{ HCO}_{\text{3}}^{\text{-}}\text{ +} \text{4H}_{\text{2}}\text{ ↔}\text{ CH}_{\text{4 }}\text{+} \text{3H}_{\text{2}}\text{O}$ | -158 | -167 |
| $\text{4CH}_{\text{3}}\text{OH }\text{↔}\text{ 3CH}_{\text{4 }}\text{+ }\text{H}^{\text{+}}\text{ }{\text{+ }\text{ HCO}_{\text{3}}^{\text{-}}\text{ + 3H}}_{\text{2}}\text{O}$ | -121 | -118 |
| $\text{4CH}_{\text{3}}\text{OH + }\text{H}_{\text{2}}\text{ }\text{↔}\text{ CH}_{\text{4 }}\text{+ }\text{H}_{\text{2}}\text{O}$ | -130 | -130 |
| 4CH­_3_­NH_2_ + 3H_2_O + 3H^+^ $\leftrightarrow$ 3CH_4_ + $\text{ HCO}_{\text{3}}^{\text{-}}$ + 4N$\text{H}_{\text{4}}^{\text{+}}$ | -136 | -134 |
| CH_3_NH_2_ + H_2_ + H^+^ $\leftrightarrow$ CH_4_ + N$\text{H}_{\text{4}}^{\text{+}}$ | -143 | -143 |
| 2(CH_3_)_2_NH + 3H_2_O + H^+^ $\leftrightarrow$ 3CH_4_ + $\text{ HCO}_{\text{3}}^{\text{-}}$ + 2N$\text{H}_{\text{4}}^{\text{+}}$ | -103 | -98 |
| (CH_3_)_2_NH + 2H_2_ + H^+^ $\leftrightarrow$ 2CH_4_ + 2N$\text{H}_{\text{4}}^{\text{+}}$ | -117 | -116 |
| 4(CH_3_)_3_N + 9H_2_O + H^+^ $\leftrightarrow$ 9CH_4_ + 3$\text{HCO}_{\text{3}}^{\text{-}}$ + 4N$\text{H}_{\text{4}}^{\text{+}}$ | -91 | -86 |
| (CH_3_)_3_N + 3H_2_ + H^+^ $\leftrightarrow$ 3CH_4_ + 3 N$\text{H}_{\text{4}}^{\text{+}}$ | -108 | -106 |
| CH_3_COO^-^ + H^+^ $\leftrightarrow$ CH_4_ + CO_2_ | -25 | -21 |

**Table S5.** Average pH of *M. barkeri* cultures.

|  | 30˚C | | 0˚C | |
| --- | --- | --- | --- | --- |
| Condition | Day 0 | Day 28 | Day 0 | Day 28 |
| Control | 7.0 | 6.51 | 7.0 | 6.81 |
| Mg(ClO_4_)_2_ | 7.2 | 6.49 | 7.2 | 6.76 |
| Na(ClO_4_) | 7.25 | 6.48 | 7.25 | 6.86 |
| Ca(ClO_4_)_2_ | 7.0 | 6.78 | 7.0 | 6.53 |

**Tables S6 – S12** can be found as additional .xlsx files.

**Table S13.** Gene abbreviations of proteins visualized in Figure 3.

| **Encoded Protein** | **Nomenclature** |
| --- | --- |
| Methanol methyltransferase corrinoid protein | *mta*C |
| Methanol:corrinoid methyltransferase | *mta*B |
| Methanol-specific methylcobalamin:coenzyme M methyltransferase | *mta*A |
| Trimethylamine methyltransferase corrinoid protein | *mtt*C |
| Trimethylamine:corrinoid methyltransferase | *mtt*B |
| Dimethylamine permease | *mtb*P |
| Dimethylamine methyltransferase corrinoid protein | *mtb*C |
| Dimethylamine:corrindoid methyltransferase | *mtb*B |
| Monomethylamine permease | *mtm*P |
| Monomethylamine methyltransferase corrinoid protein | *mtm*C |
| Monomethylamine:corrinoid protein | *mtm*B |
| Methylamine-specific methylcobalamin:coenzyme M methyltransferase | *mtb*A |
| Acetyl-CoA Synthetase | *acs* |
| Acetate kinase | *ack*A |
| Phosphoacetyl transferase | *pta* |
| Carbon monoxide dehydrogenase cooS subunit | *coo*S |
| Carbon monoxide dehydrogenase cooF subunit | *coo*F |
| CO dehydrogenase accessory protein cooC (nickel insertion) | *coo*C |
| CO dehydrogenase/acetyl-CoA subunit epsilon, CODH subcomplex | *cdh*$\varepsilon$ |
| CO dehydrogenase/acetyl-CoA synthase subunit delta, corrinoid FeS subcomplex small subunit | *cdh*$\delta$ |
| 5-H_4_SPT:corrinoid iron-sulfur protein methyltransferase | *cdh*$\gamma$ |
| CO dehydrogenase/acetyl-CoA synthase subunit beta, acetyl-CoA synthase | *cdh*$\beta$ |
| CO dehydrogenase/acetyl-CoA synthase subunit alpha, CO dehydrogenase subcomplex | *cdh*$\alpha$ |
| Methyl coenzyme M reductase system component A2 | *atw* |
| Methyl-coenzyme M reductase gamma subunit | *mcr*G |
| Methyl-coenzyme M reductase delta subunit | *mcr*D |
| Methyl-coenzyme M reductase operon protein C | *mcr*C |
| Methyl-coenzyme M reductase beta subunit | *mcr*B |
| Methyl-coenzyme M reductase alpha subunit | *mcr*A |
| Coenzyme F_420_ hydrogenase subunit gamma | *frh*$\gamma$ |
| Coenzyme F_420_ hydrogenase subunit beta | *frh*$\beta$ |
| Coenzyme F_420_ hydrogenase subunit alpha | *frh*$\alpha$ |
| Methanophenazine hydrogenase cytochrome b subunit | *vht*C |
| Methanophenazine hydrogenase small subunit | *vht*G |
| Methanopheazine hydrogenase large subunit | *vht*A |
| N5-methyl-H_4_SPT:methyltransferase, subunit H | *mtr*H |
| H_4_SPT S-methyltransferase subunit G | *mtr*G |
| H_4_SPT S-methyltransferase subunit F | *mtr*F |
| H_4_SPT S-methyltransferase subunit E | *mtr*E |
| H_4_SPT S-methyltransferase subunit D | *mtr*D |
| H_4_SPT S-methyltransferase subunit C | *mtr*C |
| H_4_SPT S-methyltransferase subunit B | *mtr*B |
| Na^+^ transporting methyl-H_4_SPT:coenzyme M methyltransferase subunit A | *mtr*A |
| F_420_-H_2_-dependent methylene-H_4_SPT reductase | *mer* |
| F_420_-dependent methylene-H_4_SPT dehydrogenase | *mtd* |
| CoB--CoM heterodisulphide reductase subunit E | *hdr*E |
| CoB--CoM heterodisulphide Fe-S subunit D | *hdr*D |
| CoB--CoM heterodisulphide reductase subunit C | *hdr*C |
| CoB--CoM heterodisulfide reductase subunit B | *hdr*B |
| CoB--CoM heterodisulfide reductase subunit A | *hdr*A |
| Methenyl-tetrahydrosarcinopterin (H_4_SPT) cyclohydrolase | *mch* |
| Formylmethanofuran-H_4_SPT formyltransferase | *ftr* |
| Energy-conserving hydrogenase (ferredoxin), subunit F | *ech*F |
| Energy-conserving hydrogenase (ferredoxin), subunit E | *ech*E |
| Energy-conserving hydrogenase (ferredoxin), subunit D | *ech*D |
| Energy-conserving hydrogenase (ferredoxin), subunit C | *ech*C |
| Energy-conserving hydrogenase (ferredoxin), subunit B | *ech*B |
| Energy-conserving hydrogenase (ferredoxin), subunit A | *ech*A |
| Polyferredoxin protein (4Fe-4S ferredoxin) | *mvh*B |
| (Mo-containing) formylmethanofuran dehydrogenase subunit G | *fmd*G |
| (Mo-containing) formylmethanofuran dehydrogenase subunit F | *fmd*F |
| (Mo-containing) formylmethanofuran dehydrogenase subunit E | *fmd*E |
| (Mo-containing) formylmethanofuran dehydrogenase subunit D | *fmd*D |
| (Mo-containing) formylmethanofuran dehydrogenase subunit C | *fmd*C |
| (Mo-containing) formylmethanofuran dehydrogenase subunit B | *fmd*B |
| (Mo-containing) formylmethanofuran dehydrogenase subunit A | *fmd*A |
| (W-containing) formylmethanofuran dehydrogenase subunit D | *fwd*D |
| (W-containing) formylmethanofuran dehydrogenase subunit G | *fwd*G |
| F_420_H_2_ dehydrogenase subunit A | *fpo*A |
| F_420_H_2_ dehydrogenase subunit B | *fpo*B |
| F_420_H_2_ dehydrogenase subunit C | *fpo*C |
| F_420_H_2_ dehydrogenase subunit D | *fpo*D |
| F_420_H_2_ dehydrogenase subunit E | *fpo*E |
| F_420_H_2_ dehydrogenase subunit F | *fpo*F |
| F_420_H_2_ dehydrogenase subunit G | *fpo*G |
| F_420_H_2_ dehydrogenase subunit H | *fpo*H |
| F_420_H_2_ dehydrogenase subunit I | *fpo*I |
| F_420_H_2_ dehydrogenase subunit J | *fpo*J |
| F_420_H_2_ dehydrogenase subunit K | *fpo*K |
| F_420_H_2_ dehydrogenase subunit L | *fpo*L |
| F_420_H_2_ dehydrogenase subunit M | *fpo*M |
| F_420_H_2_ dehydrogenase subunit N | *fpo*N |
| F_420_H_2_ dehydrogenase subunit O | *fpo*O |

**Table S14.** Abbreviated metabolites visualized in Figures 3.

| **Metabolite** | **Nomenclature** |
| --- | --- |
| Coenzyme M | CoM-SH |
| Methyl-coenzyme M | CH_3_-S-CoM |
| Coenzyme B | CoB-SH |
| Coenzyme F_420_H_2_ | F420_red_ |
| Coenzyme F_420_ | F420_ox_ |
| Reduced ferredoxin | Fd_red_ |
| Oxidized ferredoxin | Fd_ox_ |
| Methanophenazine-H_2_ | MP_red_ |
| Methanophenazine | MP_ox_ |
| Tetrahydrosarcinopterin | H_4_MPT |
| Acetyl-Coenzyme A | Acetyl-CoA |
| Methanofuran | MFR |

**Table S15**. Gene abbreviations of proteins visualized in Figure 4.

| **Encoded Protein** | **Nomenclature** |
| --- | --- |
| Ammonium transporter  Nitrogenase FeS scaffold assembly protein | *amt*  *nif*B |
| Nitrogenase FeMo protein alpha chain | *nif*D |
| Nitrogenase FeMo biosynthesis protein | *nif*E |
| Nitrogenase FeMo reductase and maturase protein | *nif*H |
| Nitrogenase regulatory protein P-II | *nif*I |
| Nitrogenase FeMo protein beta chain | *nif*K |
| Nitrogenase FeMo-cofactor scaffold and assembly protein | *nif*N |
| Nitrogenase vanadium cofactor synthesis protein | *vnf*E |
| Nitrogenase vanadium-iron protein beta chain | *vnf*K |

**Table S16.** Gene abbreviations of proteins visualized in Figure 5a.

| **Encoded Protein** | **Nomenclature** |
| --- | --- |
| Tryptophan beta chain | *trp*B |
| Threonine synthetase | *thr*C |
| Serine—glyoxylate aminotransferase | *SGAT* |
| Phosphoserine phosphatase | *ser*B |
| Aspartate carbamoyltransferase | *pyr*B |
| Pyrrolysine synthetase | *pyl*C |
| Phosphoribosylformylglycinamidine synthase, glutamine amidotransferase subunit | *pyr*Q |
| Pyrroline-5-carboxylate reductase | *pro*C |
| Prephenate dehydratase | *phe*A2 |
| Methionine gamma-lyase | *mgl* |
| 5-methyltetrahydropteroyltriglutamate—homocysteine methyltransferase | *met*E |
| Diaminopimelate decarboxylase | *lys*A |
| Branched-chain amino acid aminotransferase | *ilv*E |
| Histidinol dehydrogenase | *his*D |
| Aspartate aminotransferase / Histidinol-phosphate aminotransferase | *his*C |
| Serine hydroxymethyltransferase / L-threonine aldolase / L-allo threonine aldolase | *gly*A |
| Glutamate synthase (NADPH) small chain | *glt*D |
| Glutamine synthetase type I | *gln*A |
| Glutamate dehydrogenase 2 | *gdh*A |
| Cysteine synthase | *cys*K |
| Serine acetyltransferase | *cys*E |
| Biosynthetic aromatic amino acid aminotransferase alpha / Aspartate aminotransferase | *asp*B |
| Asparagine synthetase (glutamine hydrolyzing) | *asn*B |
| Argininosuccinate synthase | *arg*G |
| Arginine deaminase | *arc*A |
| Ornithine cyclodeaminase | *ala* |
| Phosphoserine aminotransferase | *ser*C |
| D-3-phosphoglycerate dehydrogenase | *ser*A |
| 5'-methylthioadenosine phosphorylase | *mtn*N |
| Methylthioribose-1-phosphate isomerase | *mtn*A |
| S-adenosylhomocysteine deaminase | *mta*D |
| Homoserine O-acetyltransferase | *met*X |
| Archaeal S-adenosylmethionine synthetase | *met*K |
| O-acetylhomoserine sulfhydralase | *met*C |
| Malade dehydrogenase | *mdh* |
| Aspartokinase | *lys*C |
| Homoserine dehydrogenase | *hsd* |
| Ferredoxin | *fdx* |
| Aspartate aminotransferase | *asp*C |
| Aspartate-semialdehyde dehydrogenase | *asd* |
| Adenosylhomocysteinase | *ach*Y |

**Table S17.** Amino acid abbreviations reported from Figure 5b.

| **Amino Acid** | **Nomenclature** |
| --- | --- |
| Alanine | A |
| Arginine | R |
| Asparagine | N |
| Aspartate | D |
| Cysteine | C |
| Glutamine | Q |
| Glutamate | E |
| Glycine | G |
| Histidine | H |
| Isoleucine | I |
| Lysine | K |
| Methionine | M |
| Phenylalanine | F |
| Proline | P |
| Pyrrolysine^a^ | O |
| Serine | S |
| Threonine | T |
| Tryptophan | W |
| Tyrosine | Y |
| Valine | V |

^a^Pyrrolysine is a lysine derivative encoded by the UAG codon in *Methanosarcina barkeri*^1^.

**REFERENCES**

1. Srinivasan, G. Pyrrolysine Encoded by UAG in Archaea: Charging of a UAG-Decoding Specialized tRNA. *Science* **296**, 1459–1462 (2002).
